# Triggerable Protocell Capture in Nanoparticle-Caged Coacervate Microdroplets

**DOI:** 10.1021/jacs.1c11414

**Published:** 2022-02-22

**Authors:** Ning Gao, Can Xu, Zhuping Yin, Mei Li, Stephen Mann

**Affiliations:** † Max Planck-Bristol Centre for Minimal Biology, University of Bristol, Bristol BS8 1TS, U.K.; ‡ Centre for Protolife Research and Centre for Organized Matter Chemistry, School of Chemistry, University of Bristol, Bristol BS8 1TS, U.K.; § School of Materials Science and Engineering, Shanghai Jiao Tong University, Shanghai 200240, P. R. China

## Abstract

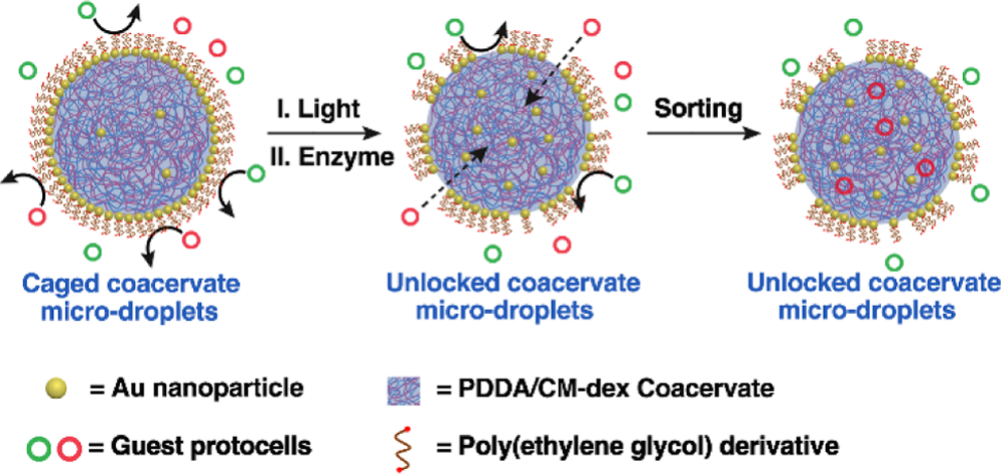

Controlling the dynamics of mixed communities of cell-like entities (protocells) provides a step toward the development of higher-order cytomimetic behaviors in artificial cell consortia. In this paper, we develop a caged protocell model with a molecularly crowded coacervate interior surrounded by a non-cross-linked gold (Au)/poly(ethylene glycol) (PEG) nanoparticle-jammed stimuli-responsive membrane. The jammed membrane is unlocked by either exogenous light-mediated Au/PEG dissociation at the Au surface or endogenous enzyme-mediated cleavage of a ketal linkage on the PEG backbone. The membrane assembly/disassembly process is used for the controlled and selective uptake of guest protocells into the caged coacervate microdroplets as a path toward an all-water model of triggerable transmembrane uptake in synthetic protocell communities. Active capture of the guest protocells stems from the high sequestration potential of the coacervate interior such that tailoring the surface properties of the guest protocells provides a rudimentary system of protocell sorting. Our results highlight the potential for programming surface-contact interactions between artificial membrane-bounded compartments and could have implications for the development of protocell networks, storage and delivery microsystems, and microreactor technologies.

## Introduction

Synthetic cell research offers diverse opportunities to carry out compartmentalized biomimetic reactions, unravel the foundations of living systems, and promote the development of bottom-up synthetic biology.^1−7^ At the population-level, controlling the dynamics of mixed communities of synthetic cells by contact-dependent interactions provides steps toward the development of higher-order cytomimetic behaviors such as artificial predation,^8,9^ parasitism,^10^ and phagocytosis.^11,12^ Although a range of water-based membrane-bounded protocell models are currently available, the use of cross-linked membrane building blocks in colloidosomes,^13^ proteinosomes,^14^ and polysaccharidosomes^15,16^ and the restricted membrane dynamics of lipid vesicles^17^ and polymersomes^18^ limit the possibilities for transmembrane transport and trafficking of cell-sized objects in synthetic cell consortia. Moreover, as these model systems are close to equilibrium, there is minimal driving force for active colloidal transport even if pores of sufficient size could be established within the constituent membranes of synthetic cells. These challenges have been recently addressed using polymerized siloxane vesicles comprising a single membrane aperture that functions as a size-selective pore for the capture and expulsion of colloidal particles using an encapsulated phoretic pump to achieve non-equilibrium conditions.^19^

**Figure 1:**
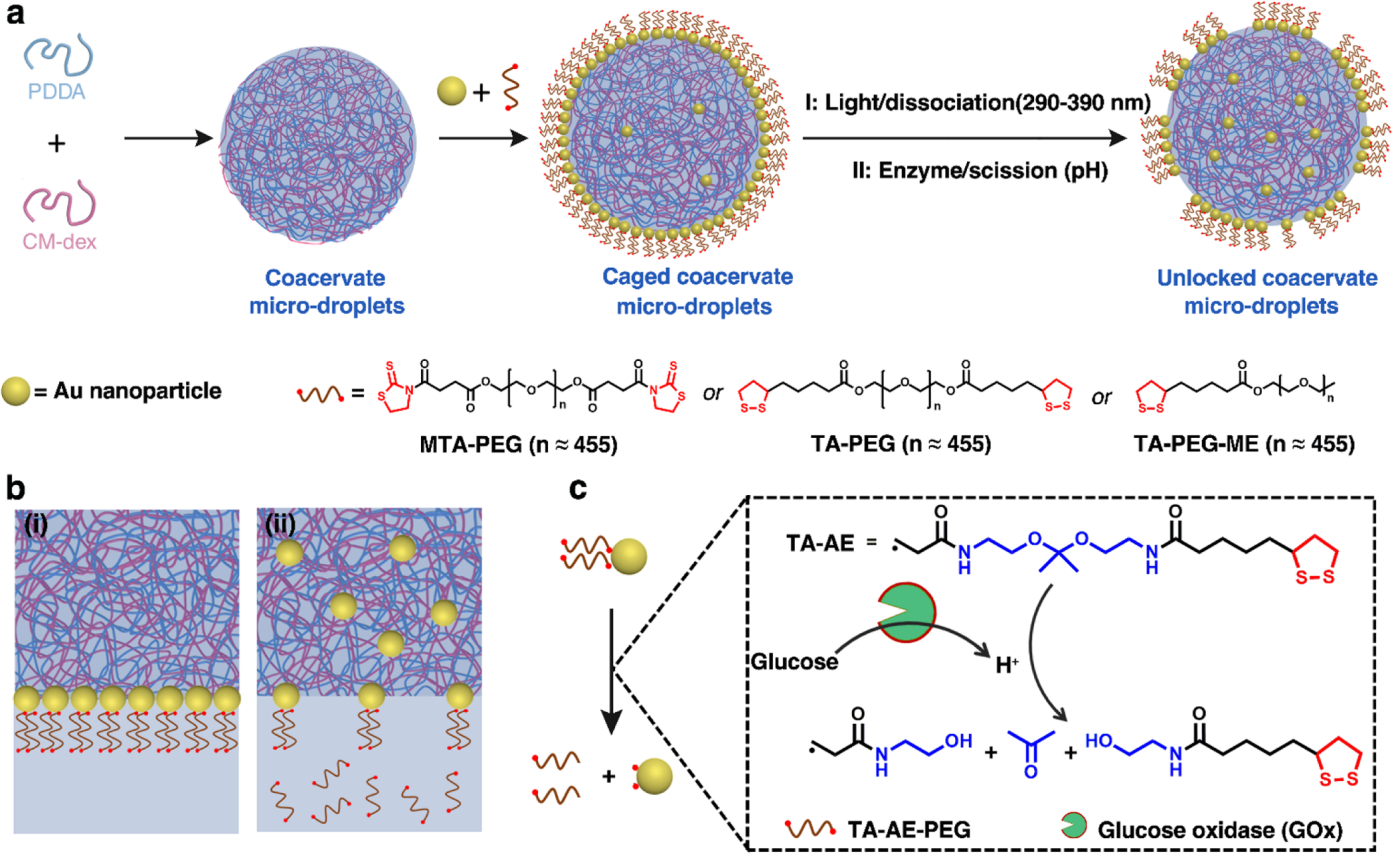
Construction of nanoparticle-caged coacervate protocells and membrane unlocking. (a) Scheme showing self-assembly and triggered membrane dynamics in caged PDDA/CM-dex coacervate droplets. Tannic-acid-coated Au nanoparticles are rendered amphiphilic at the water/coacervate droplet interface by asymmetric ligand exchange with bidentate ligands TA-PEG, MTA-PEG, or TA-AE-PEG6k or monodentate TA-PEG-ME to produce a membrane of jammed Au/PEG Janus-like nanoparticles that can be unlocked by exogenous light-mediated dissociation (I; Au/TA-PEG, Au/MTA-PEG, and Au/TA-PEG-ME) or endogenous enzyme-mediated molecular cleavage (II; TA-AE-PEG6k). (b) Graphics showing proposed models of the jammed (i) and unjammed (ii) membranes; light- or chemically induced ligand dissociation results in translocation of PEG-depleted Au nanoparticles into the coacervate matrix and formation of apertures in the membrane. (c) Mechanism of glucose oxidase (GOx)/glucose-mediated ligand dissociation from Au/TA-AE-PEG6k nanoparticles. Addition of glucose in the presence of dioxygen generates gluconic acid within the caged protocell, which cleaves TA-AE-PEG6k and unlocks the membrane.

Herein, we present an alternative strategy in which light- or chemical-mediated relaxation of the structurally stressed membrane of a host protocell facilitates the all-water capture of guest protocells located in the external environment. Our approach is based on the development of a new protocell model derived from the surface augmentation of coacervate microdroplets. Coacervates have been previously employed as membrane-less protocell models with a range of cytomimetic advantages including the partitioning of biological molecules and machinery,^20−23^ modulation of protein folding/unfolding, association and diffusion,^24−26^ rate enhancement of gene expression and mRNA production,^27−29^ and promotion of dissipative protein self-assembly.^30^ Significantly, the molecularly crowded milieu provides an interfacial barrier in water that facilitates the spontaneous capture of microscale objects by contact-dependent interactions,^10^ offering a potential driving force for colloidal transport in membrane-bounded coacervate protocells furnished with triggerable membrane dynamics. In this context, although membranized coacervate droplets have been prepared using molecular amphiphiles (fatty acids,^31^ block copolymers,^32,33^ and phospholipids^17,34,35^), surface complexation agents [polyoxometalates (POMs)^36^ and sodium dodecyl sulfate,^37^] and surface-active colloids (modified proteins, and red blood or yeast cell membrane fragments^38−40^), the boundary layers are structurally persistent and unresponsive to the uptake of external microscale objects. To address this challenge, we develop a membrane construction process based on the spontaneous monolayer assembly and non-equilibrium locking of stimuli-responsive Janus-like ligated gold (Au) nanoparticle surfactants at the water/coacervate droplet interface (Figure 1a). As nanoparticle surfactants typically form in situ at oil/water boundaries and comprise stimuli-responsive polymers for unjamming the membrane,^41−43^ we prepared novel nanoparticle constructs specifically for reversible stabilization of the water/coacervate droplet interface. Consequently, we describe a new type of the caged protocell model with a molecularly crowded interior surrounded by a non-cross-linked structurally stressed membrane of jammed Au nanoparticles. Critically, the membrane can be unlocked by ligand dissociation arising exogenously from light illumination at the Au surface^44^ or endogenously via enzyme-mediated cleavage of a ketal linkage (Figure 1b,c). We exploit the light- or chemical-mediated triggering of apertures in the Au nanoparticle membrane to selectively capture external cell-like objects within the coacervate interior, providing opportunities to implement mechanisms of functional integration (symbiosis), sorting, and logistics in synthetic cell communities.

## Results and Discussion

### Membrane Assembly and Unjamming in Caged Coacervate Protocells

**Figure 2:**
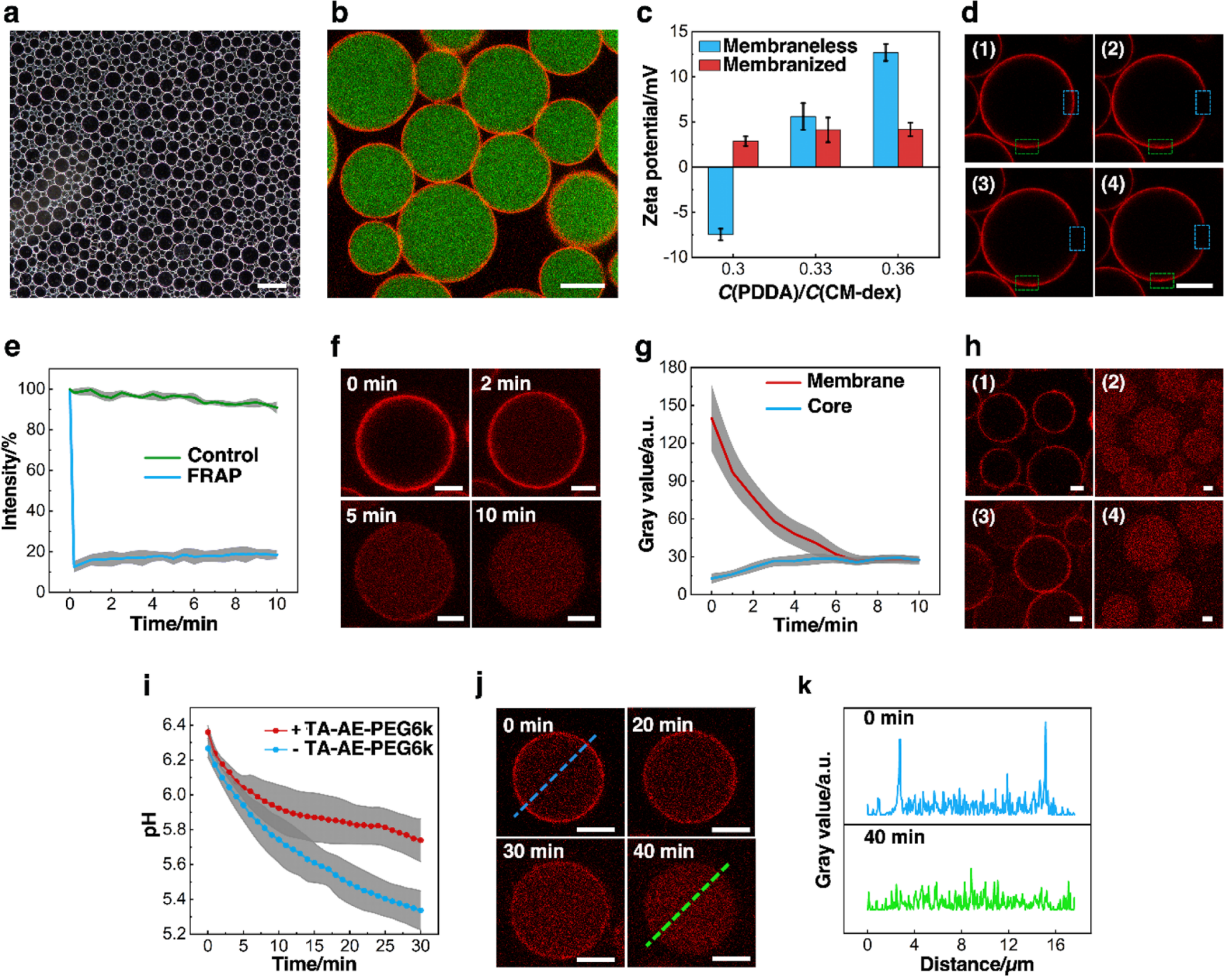
Caging and unlocking of coacervate protocells. (a) Dark field microscopy image showing multilayer population of nanoparticle-caged coacervate microdroplets. (b) Confocal laser scanning microscopy (CLSM) image of caged coacervate droplets; red fluorescence membrane (Au/RITC-labeled TA-PEG nanoparticles); and green fluorescence interior (FITC-labeled CM-dex coacervate). (c) Zeta-potential measurements showing variation of coacervate droplet surface charge before and after Au/MTA-PEG membranization. Samples were prepared at a range of PDDA/CM-dex molar ratios. (d) Time series of CLSM images of Au-nanoparticle-caged coacervate droplets recorded before (1), 30 s after photobleaching (2), 5 min after recovery (3), and 10 min after recovery (4). The photobleaching and control areas are delineated by blue or green rectangles, respectively. Red fluorescence, Au/RITC-labeled TA-PEG nanoparticles. (e) Plots of changes in fluorescence intensity for delineated areas shown in (d). Minimal recovery of the fluorescence is observed over 10 min due to the solid-like membrane. (f,g) Time series of CLSM images (f) and corresponding changes in membrane and interior (core) red fluorescence [(g) gray value] for an Au/RITC-TA-PEG nanoparticle-caged coacervate droplet after light illumination. (h) CLSM images of Au/RITC-labeled TA-PEG nanoparticle-caged coacervate droplets before (1) and after (2) exposure to light showing membrane disassembly and translocation of the de-capped Au nanoparticles into the coacervate interior. Application of a shear force reassembles the membrane (3), which can be subsequently disassembled by further light exposure (4). (i) Plot showing time-dependent decreases in pH associated with glucose-triggered GOx activity in Au/TA-AE-PEG6k-caged coacervate droplets (+) or membrane-less coacervate droplets containing GOx (−). The pH decrease is lower in the presence of TA-AE-PEG6k due to the consumption of protons in the cleavage reaction. GOx, glucose, and TA-AE-PEG6k concentrations are 0.2 mg/mL, 10 mM, and 0.2 mg/mL, respectively. (j) Time series of CLSM images of a single GOx-containing Au/RITC-labeled-TA-AE-PEG6k-caged coacervate droplet before and after addition of glucose showing chemically mediated membrane disassembly. (k) Corresponding fluorescence intensity (gray values) profiles of the caged coacervate droplet shown in (j), before (top) and 40 min after (bottom) addition of glucose. Scale bars: 50 (a), 10 (b), and 5 μm (d,f,h,j). Error bars represent the standard deviation; (c,e,g,i), *n* = 3, 3, 20, and 3, respectively.

Coacervate microdroplets were produced by mixing aqueous solutions of poly(diallyldimethyl ammonium chloride) (PDDA) and carboxymethyl-dextran (CM-dex) at a molar ratio of 4:9 (Figure S1). Spontaneous membranization of the PDDA/CM-dex coacervate droplets was accomplished by addition of tannic acid-protected Au nanoparticles (5–7 nm in diameter, Figure S2) followed within a few seconds by (±)-1,2-dithiolane-3-pentanoic acid (thioctic acid)-modified polyethylene glycol (TA-PEG, *M*_w_ = 20 kDa, *n* ≈ 455; Figure 1a) under vigorous stirring to produce nanoparticle-caged coacervate droplets, ca. 30 μm in mean diameter (Figures 2a,b, S3 and S4). Similar results were obtained using 2-mercapto-2-thiazoline-modified PEG (MTA-PEG; Figure 1a) (Figure S5), a monodentate TA-modified PEG (TA-PEG-ME, Figures 1a and S6), or a TA-PEG derivative with a decreased molecular weight (6 kDa) and acid-cleavable ketal linker (TA-AE-PEG6k; AE = 2,2-bis(aminoethoxy)propane; Figures 1c and S7) in place of TA-PEG. In general, stabilized droplets were produced under a limited range of molar ratios with insufficient or excess TA-PEG resulting in Au/TA-PEG sequestration or exclusion, respectively (Figures S8 and S9). In addition, the caged protocells were not formed when the order of reagent addition was reversed, or both components were added simultaneously. As the tannic acid-capped Au nanoparticles alone were readily sequestered by the coacervate droplets but excluded from the droplets when coacervate-insoluble TA-PEG was added first to homogeneously replace the tannic acid (Figure S10), we attributed localization of the Au/TA-PEG building blocks to the in situ formation of a low-charge, Janus-like nanoparticle surfactant at the droplet/water interface. This was consistent with zeta-potential measurements that showed a constant near-neutral surface charge for the caged coacervate droplets (Figure 2c).

Under ambient conditions, the self-assembled Au/TA-PEG membrane exhibited high permeability to small molecules, polyelectrolytes, and nanosized objects such as proteins [bovine serum albumin (BSA), amylase, and GOx] and polysaccharides (Figure S11). In general, the solutes did not bind strongly to the Au/TA-PEG membrane, presumably due to the steric barrier accompanying in situ pegylation of the amphiphilic nanoparticles but were partitioned and concentrated within the internal coacervate matrix. In contrast, micrometer-sized objects in the external environment were excluded from the caged coacervate droplets.

We investigated the light-mediated membrane dynamics of the Au/TA-PEG and Au/MTA-PEG-caged coacervate droplets by irradiating the samples at 290–390 nm for several minutes. Fluorescence recovery after photobleaching (FRAP) experiments indicated that prior to light-induced dissociation, the Au/TA-PEG nanoparticle surfactants were packed into a solid-like membrane (Figure 2d,e), while the coacervate phase was liquid-like (Figure S12). Replacing TA-PEG with monodentate TA-PEG-ME also gave a solid-like membrane, indicating that bidentate ligand-mediated cross-linking was not responsible for stabilization of the Au nanoparticle shell (Figure S13). The absence of ligand cross-linking was consistent with other observations, which revealed that dissolving the coacervate interior of Au/TA-PEG-caged droplets by slow addition of distilled water resulted in concomitant disassembly of the nanoparticle membrane (Figure S14). The jammed membrane was relaxed by light-driven TA-PEG disassociation and release, resulting in progressive translocation of the partially de-capped Au nanoparticles into the coacervate interior and slow disassembly of the outer membrane over 10 min (Figures 2f,g and S15). We attributed the light-mediated dissociation at 290–390 nm to coupling between photoexcited electrons of the Au nanoparticles and gold–sulfur bond vibrations,^44^ rather than a photothermal effect associated with the surface plasmon resonance (SPR) band, which is also known to cleave the gold–sulfur bond.^45^ The photothermal mechanism was ruled out as illumination of the caged droplets at 500–520 nm (Au nanoparticle SPR band) did not result in membrane disassembly (Figure S16).

Re-assembly of the nanoparticle membrane was accomplished by switching off the light and using a shear force to drive the sequestered Au nanoparticles back to the coacervate/water interface to initiate rebinding with TA-PEG (Figure 2h). The reversibility was limited to two cycles as dissociation of the Au/TA-PEG conjugate gradually resulted in non-specific nanoparticle aggregation.

Membrane unlocking was also achieved under ambient daylight by chemically induced ligand cleavage and dissociation using GOx-containing caged coacervate droplets enclosed in a jammed shell of Au/TA-AE-PEG6k nanoparticle surfactant building blocks. Cleavage of the ketal group at different pH values was quantitatively determined by NMR spectroscopy (Figure S17). Minimal bond cleavage was observed at pH values higher than 6.5, while decreasing the pH to 5.3 completely hydrolyzed the ketal linkage within 60 min. Addition of glucose to the external solution of a suspension of GOx-containing caged droplets in the presence of dioxygen resulted in a decrease in pH from 6.4 to 5.8 over 30 min (Figure 2i) and progressive relaxation of the Au/TA-AE-PEG6k membrane. The jammed membrane was typically unlocked within ca. 30–40 min after addition of glucose (Figure 2j,k). No membrane disassembly was observed in control experiments undertaken in the absence of GOx and glucose (Figure S18).

### Triggerable Protocell Capture in Caged Coacervate Microdroplets

**Figure 3:**
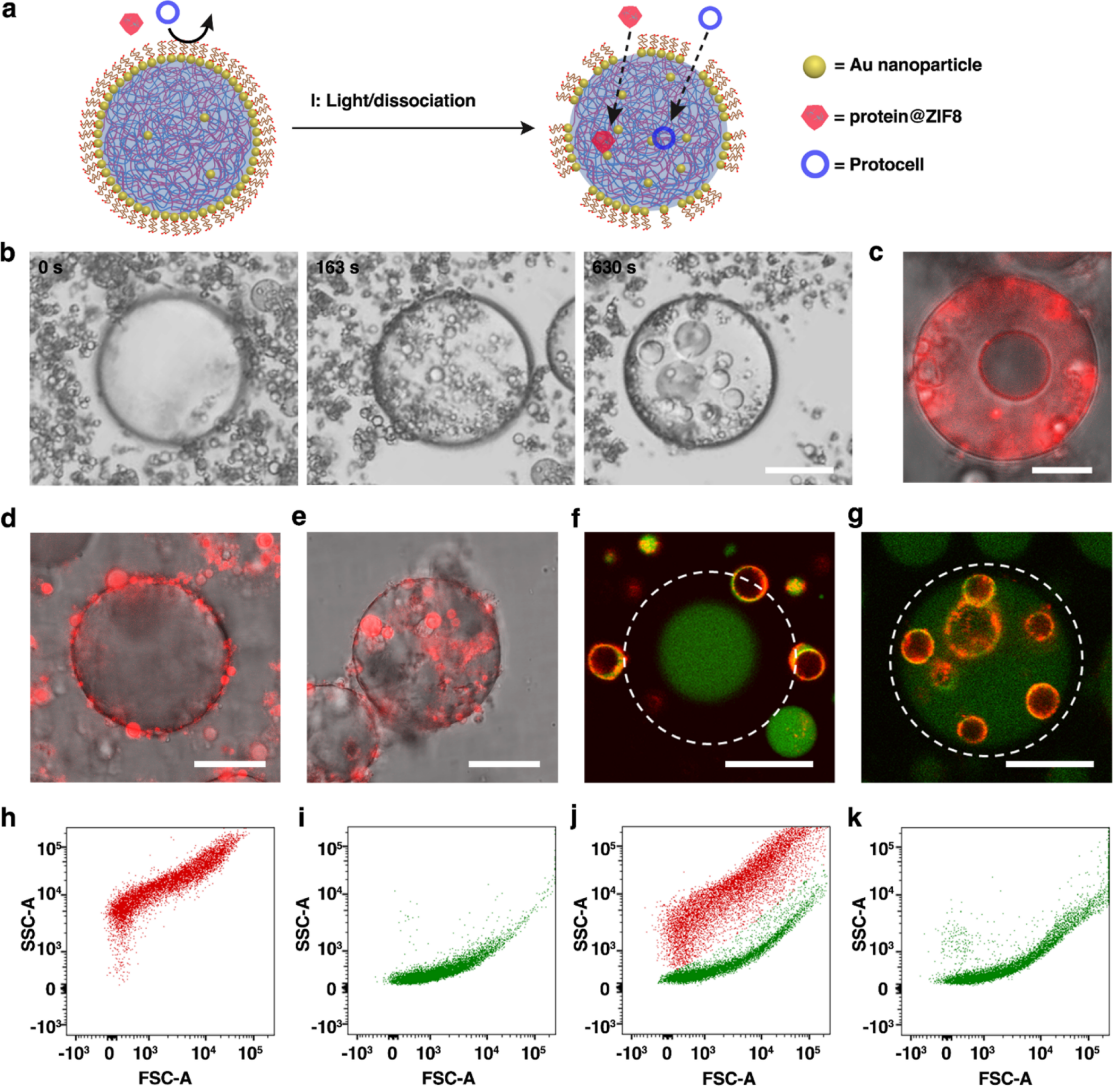
Stimuli-responsive uptake and capture in nanoparticle-caged coacervate microdroplets. (a) Scheme showing light-mediated relaxation of mechanical strain and appearance of membrane apertures in Au/TA-PEG nanoparticle-caged coacervate droplets, leading to the uptake and capture of external microparticles (protein@ZIF8; mean size, 0.5 μm; red) or guest protocells (PCVs; mean diameter, 4 μm; blue). (b) Time series of optical images of an individual caged coacervate droplet surrounded by a dense population of PCVs recorded before (left) and after 163 s (middle) and 630 s (right) of continuous light exposure. The PCVs are progressively taken up and captured within the unjammed caged coacervate droplets. Samples were mounted onto a pegylated glass substrate. (c) Overlay of fluorescence and bright field images of an individual caged coacervate droplet and RITC-labeled PCVs (red fluorescence) recorded 10 min after continuous exposure to light at 290–390 nm. (d,e) Overlays of fluorescence and bright field images of an individual caged coacervate droplet and RITC-labeled colloidosomes (red fluorescence) recorded in ambient daylight (d) and 10 min after continuous exposure to light at 290–390 nm (e) showing transformation from non-interacting protocells to contact-dependent transmembrane transfer. Scale bars, 20 μm. (f,g) CLSM images of red/green fluorescence overlay images showing a single FITC-labeled GOx-containing Au/TA-AE-PEG6k-caged coacervate droplet surrounded by RITC-labeled PCV and recorded before (f) and 60 min after (g) addition of glucose. Unjamming of the membrane by enzyme-mediated cleavage of TA-AE-PEG6k results in PCV transfer across the membrane. The focal plane is aligned with the PCVs not the caged coacervate droplet. White dash circles delineate the boundary of the caged coacervate droplet. Scale bars, 20 μm. (h–k), FACS-derived 2D dot plots of side-scattered light (SSC) vs forward-scattered light (FSC) for single population of PCVs (h), single population of GOx-loaded caged droplets (i), mixture of PCVs and GOx-loaded caged droplets in the absence of glucose (j) and 1 h after the addition of glucose (k).

We used the light-induced unlocking of the structurally stressed Au/TA-PEG nanoparticle membrane as an exogenous mechanism for triggering the uptake of external microscale objects (Figure 3a). As an initial test, an aqueous dispersion of caged protocells and fluorescently labeled catalytic microparticles [RITC-BSA/zeolitic imidazolate framework (ZIF8); BSA@ZIF8, Figure S19] was irradiated at 290–390 nm. Unlocking of the membrane occurred increasingly over 6 min, giving rise to translocation of the partially de-capped Au nanoparticles into the PDDA/CM-dex coacervate interior and progressive uptake of the catalytic microparticles (Figures S20 and S21). Given these observations, we used a similar procedure to trigger the contact-dependent uptake and capture of POM-coated PDDA/ATP coacervate vesicles (PCVs; mean diameter, 4 μm; Figure S22),^36^ within the nanoparticle-caged coacervate droplets. Under ambient daylight, the two populations of membrane-bounded protocells remained non-interactive (Figure S23), while the membrane-less PDDA/CM-dex coacervate droplets spontaneously engulfed the smaller PCVs (Figure S24). Switching on the light source for approximately 10 min transferred considerable numbers of the PCVs through the partially unlocked Au/TA-PEG nanoparticle membrane (Figure 3b,c and Movie 1). The resulting host–guest membranized coacervate droplets remained structurally stable under ambient conditions provided that the illumination was not prolonged such that complete disassembly of the Au nanoparticle membrane occurred. Once trapped, the guest PCVs underwent a process of slow coalescence (Figures 3b and S25), which was attributed to the destabilization of the POM/PDDA membrane in the presence of the PDDA-enriched matrix of the host protocells.

As transfer of the PCVs across the unlocked nanoparticle membrane was facilitated by attractive interactions with the exposed PDDA/CM-dex coacervate phase, we used the combination of light-mediated membrane relaxation and favorable coacervate partitioning to trigger the uptake and capture of other types of protocells (colloidosomes, mean diameter, 5 μm) (Figures 3d,e and S26) and polymer nanoparticles (polystyrene, mean diameter, 30 nm; Figure S27). In general, provided there was an appropriate size mismatch and low interfacial tension between the host coacervate matrix and the guest protocell membrane, exposure of the mixed populations resulted in spontaneous transmembrane colloidal transfer.

As an alternative strategy, we used the endogenous GOx-mediated production of protons to trigger membrane unlocking and uptake of PCVs from the external environment. Without glucose, mixed populations of Au/TA-AE-PEG6k-caged coacervate droplets and PCVs remained non-interactive (Figure 3f). In contrast, switching on the enzyme-mediated production of gluconic acid within the coacervate phase resulted in effective capture and retention of the PCVs (Figures 3g and S28). This was confirmed by fluorescence-activated cell sorting (FACS) analysis. Profiles for single populations of RITC-labeled PCVs (Figure 3h) and FITC-labeled GOx-containing caged coacervate droplets (Figure 3i) showed different 2D dot plots of forward-scattered (FSC) versus side-scattered (SSC) light. Consequently, mixing the populations in the absence of glucose gave two distinguishable profiles (Figure 3j). Significantly, only the population of unjammed caged coacervate droplets was observed after addition of glucose (Figure 3k).

### Protocell Sorting by Nanoparticle-Caged Coacervate Microdroplets

**Figure 4:**
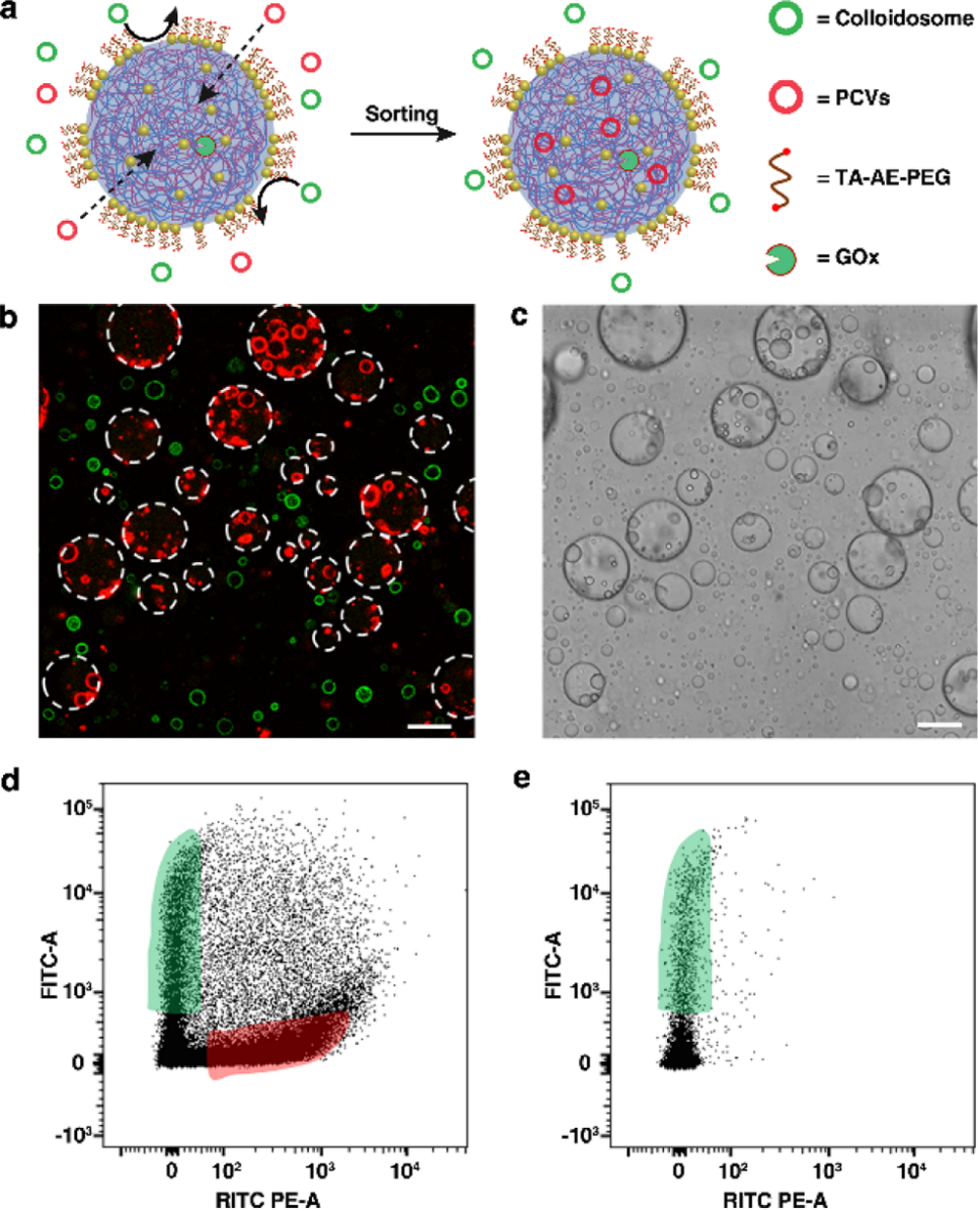
Protocell sorting by nanoparticle-caged coacervate microdroplets. (a) Scheme showing chemical-mediated protocell sorting. PCVs (red) and PEG-grafted colloidosomes (green) are mixed with GOx-loaded Au/TA-AE-PEG6k-caged coacervate droplets in the presence of glucose. Chemical-mediated membrane unlocking leads to the selective uptake and capture of only the PCVs. (b,c) CLSM image of red/green fluorescence overlay (b) and bright-field image (c) showing selective uptake of RITC-labeled PCVs (red fluorescence) into the unlocked caged coacervate droplets, while FITC-labeled PEG-tagged colloidosomes (green fluorescence) remain in the external water environment. White dash circles indicate the boundary of the unlocked caged droplets. Scale bars, 20 μm. (d) FACS-derived dot plots showing RITC (PE) fluorescence vs FITC fluorescence for a mixture of FITC-labeled PEG-tagged colloidosomes (green shading) and RITC-labeled PCVs (red shading) (volume ratio = 1:1). (e) Same as for (d) but after protocell sorting showing presence of only FITC-labeled PEG-tagged colloidosomes (green shading) in the resulting supernatant phase.

As stimuli-responsive uptake into the unjammed caged coacervate droplets was dependent on attractive interactions between the exposed PDDA/CM-dex coacervate core and outer surface of the PCVs or colloidosomes, we reasoned that colloidal transfer would be inhibited even in the presence of unlocked droplets if repulsive interactions became dominant by appropriate chemical modification of the guest protocell membrane. Thus, it should be possible to establish a mechanism of triggerable protocell sorting based on the selective uptake by unlocking the caged coacervate droplets in the presence of mixed protocell populations endowed with different affinities for the exposed PDDA/CM-dex coacervate phase. As a proof-of-principle, we used a ternary community comprising mixtures of GOx-containing Au/TA-AE-PEG6k-caged coacervate droplets as the host system and RITC-labeled PCVs and FITC-labeled PEG-grafted colloidosomes as cell-like objects capable of attractive or repulsive interactions with the PDDA/CM-dex coacervate phase, respectively (Figure 4a). Addition of glucose resulted in the selective uptake of the PCVs, while the PEG-coated colloidosomes remained dispersed in the external aqueous phase (Figure 4b,c). As a result, the colloidosomes could be separated from the mixture and collected from the low-density upper water phase of the suspension. The selective uptake and sorting process were verified by FACS analysis using an aqueous mixture of RITC-labeled PCVs and FITC-labeled PEG-tagged colloidosomes along with the caged coacervate droplets. The co-existence of both types of protocells in the ternary community was clearly detected before unjamming of the caged coacervate droplets (Figure 4d). In contrast, only green-fluorescent colloidosomes were detected in the collected supernatant phase after enzyme-mediated unlocking of the nanoparticle surfactant membrane (Figure 4e), consistent with the selective uptake of the PCVs into the coacervate droplets.

## Conclusions

A new protocell model based on the membranization of coacervate microdroplets with a jammed monolayer of stimuli-responsive Janus-like ligated Au/PEG nanoparticle surfactants is described and utilized for the contact-dependent selective capture of guest protocells in an all-aqueous environment. The structurally stressed membrane is unlocked by ligand dissociation using an exogenous light source or by chemical cleavage of the nanoparticle surfactant by enzyme-mediated transformations within the protocells. As a consequence, exposure of the coacervate interior to the external environment leads to contact-dependent sequestration of selective external colloidal objects that exhibit attractive interactions with the liquid–liquid phase-separated phase.

In general, the combination of triggerable membrane dynamics and concomitant presentation of the molecularly crowded interior offers opportunities to design elaborate protocell networks that implement complex cytomimetic behaviors such as artificial symbiosis, networking, and sorting. In principle, such higher-level behaviors could be coupled to diverse endogenous functions associated with coacervate-based protocell models,^24−30^ thereby generating artificial cell communities that operate across a range of length scales. This approach offers a system view of protocell design, providing a route to population-based synergistic behaviors such as collective resilience to environmental stress, multiplex tasking, and functional collaboration and specialization. In a wider context, triggerable collective interactivity between compartmentalized microscale objects could provide a step toward new applications in biomimetic storage and delivery and advanced microreactor technology.

## Supporting Information Available

Experimental materials and methods, ^1^H NMR spectra, TEM images, and microscopy images (PDF)
Transferring considerable numbers of the PCVs through the partially unlocked Au/TA-PEG nanoparticle membrane (MP4)
