# Supplementary material for: Triggerable Protocell Capture in Nanoparticle-Caged Coacervate Microdroplets
Source: J Am Chem Soc. 2022 Feb 22;144(9):3855–62. doi: 10.1021/jacs.1c11414 (PMC9097475; doi:10.1021/jacs.1c11414)
Supplement: Supplementary file 1 — ja1c11414_si_001.pdf [file ja1c11414_si_001.pdf]

Triggerable protocell capture in nanoparticle-caged coacervate micro-droplets

*Ning Gao [ab], Can Xu [b], Zhuping Yin [b], Mei Li [bc], Stephen Mann [abc]**
[a]Max Planck-Bristol Centre for Minimal Biology, University of Bristol, Bristol, BS8 1TS, UK
[b]Centre for Protolife Research and Centre for Organized Matter Chemistry, School of Chemistry, University of Bristol, Bristol, BS8 1TS, UK
[c]School of Materials Science and Engineering, Shanghai Jiao Tong University, Shanghai, 200240, P. R. China.
**Email: s.mann@bristol.ac.uk*

The Supporting information includes Experimental Materials and Methods, Description of the Movie, and Additional Figures.

Part 1. Supplementary materials and methods (Page 2-Page 11)
Part 2. Description of the movie (Page 12)
Part 3. Supplementary Figures (Page 13-Page 30)

Part 1. Supplementary Materials and Methods

Materials

The following chemicals were purchased from Sigma-Aldrich and used as received: Gold (III) chloride trihydrate, sodium citrate, tannic acid, potassium carbonate (K_2CO_3). Gold nanoparticles (5nm diameter, stabilized suspension in citrate buffer), poly(ethylene glycol) (average Mn = 2k, 6k, 8k, 10k, 20k), Poly(ethylene glycol) methyl ether (average Mn=20k), fluorescein isothiocyanate-dextran (average Mn= 4k and 40k), thioctic acid (TA), 4-dimethylaminopyridine (DMAP), N,N'-dicyclohexylcarbodiimide (DCC), triethylamine (TEA), 4-toluenesulfonyl chloride (TsO), sodium azide, anhydrous dichloromethane, anhydrous chloroform, dimethylformamide (DMF), N-(3-dimethylaminopropyl)-N′-ethylcarbodiimide hydrochloride (EDC), dimethyl sulfoxide (DMSO), triphenylphosphine, succinic anhydride, N-hydroxysuccinimide (NHS), 2-mercapto-2-thiazoline (MTA), 2,2-bis(aminoethoxy)propane (AE), poly(diallyldimethylammonium chloride) solution (PDDA, medium molecular weight, 20 wt% solution), carboxymethyl-dextran sodium salt (CM-dex), adenosine 5′-triphosphate disodium salt hydrate (ATP), phosphotungstic acid hydrate (PTA), carboxylate-modified polystyrene latex beads (fluorescent-labelled), tetramethoxysilane, glucose, glucose oxidase from *Aspergillus niger* (GOx, 100k-250k unit/g), α-amylase from *Aspergillus oryza*e (Amy, 150 unit/g), bovine serum albumin (BSA), fluorescein isothiocyanate isomer I (FITC), rhodamine B isothiocyanate (RITC). 2-methylimidazole and zinc nitrate hexahydrate, sodium sulfate (Na_2SO_4), sodium carbonate (Na_2CO_3) and sodium hydrogen carbonate ($NaHCO_3$) were purchased from Alfa Aesar. Coverslips were purchased from VWR International. 3-[methoxy(polyethyleneoxy)propyl]trimethoxysilane was purchased from ABCR Gute Chemie. Partial hydrophobic silica nanoparticle was purchased from Wacker chemistry.

Preparation and characterization of Au nanoparticles

Au nanoparticles were synthesized by the following protocol. 100 µL $HAuCl_4$ aqueous solution (100 mg/mL) was added to 80 mL deionized water and heated to 60 °C. 0.049 g sodium citrate, 0.01 g tannic acid and 0.0036 g K_2CO_3 were quickly added into the aqueous solution and the aqueous mixture was allowed to react under magnetic stirring (400 rpm) at 60 °C for 10 min to afford a Au-nanoparticle dispersion. The reaction was undertaken in a 250 mL round-bottom flask, which was cleaned with freshly prepared aqua regia before usage. The prepared Au-nanoparticle suspension was used without further purification.

Transmission electron microscopy (TEM) was used to characterize the morphology and diameter of the as-synthesized Au nanoparticles. UV-vis spectroscopy was used to determine the concentration of the as-synthesized Au nanoparticles. A commercial Au-nanoparticle suspension (*Sigma-Aldrich*, product number 741949, concentration $5.5 \pm 0.5 \times 10^{13}$ particles/mL) was used as a standard solution. The maximum absorption values between 500 and 550 nm of the home-synthesized and commercial Au-nanoparticle solutions were set to 0.712 (A_{510}) and 0.715 (A_{523}, diluted to 69% of the original concentration), respectively, so that the concentration of the as-synthesized Au-nanoparticle solution used was *ca*. 3.8×10^{13} particles/mL.

Synthesis of MTA-PEG, TA-PEG, RITC-PEG-TA and TA-AE-PEG

The synthetic route is shown below (Scheme S1). Poly(ethylene glycol) (PEG) with different average molecular weights (2k, 6k, 8k, 10k, 20k; denoted as PEG**n**k) was used; in theory, these correspond to ^{1}H-NMR integral numbers of the PEG main chain of 181, 545, 727, 909 and 1818, respectively.

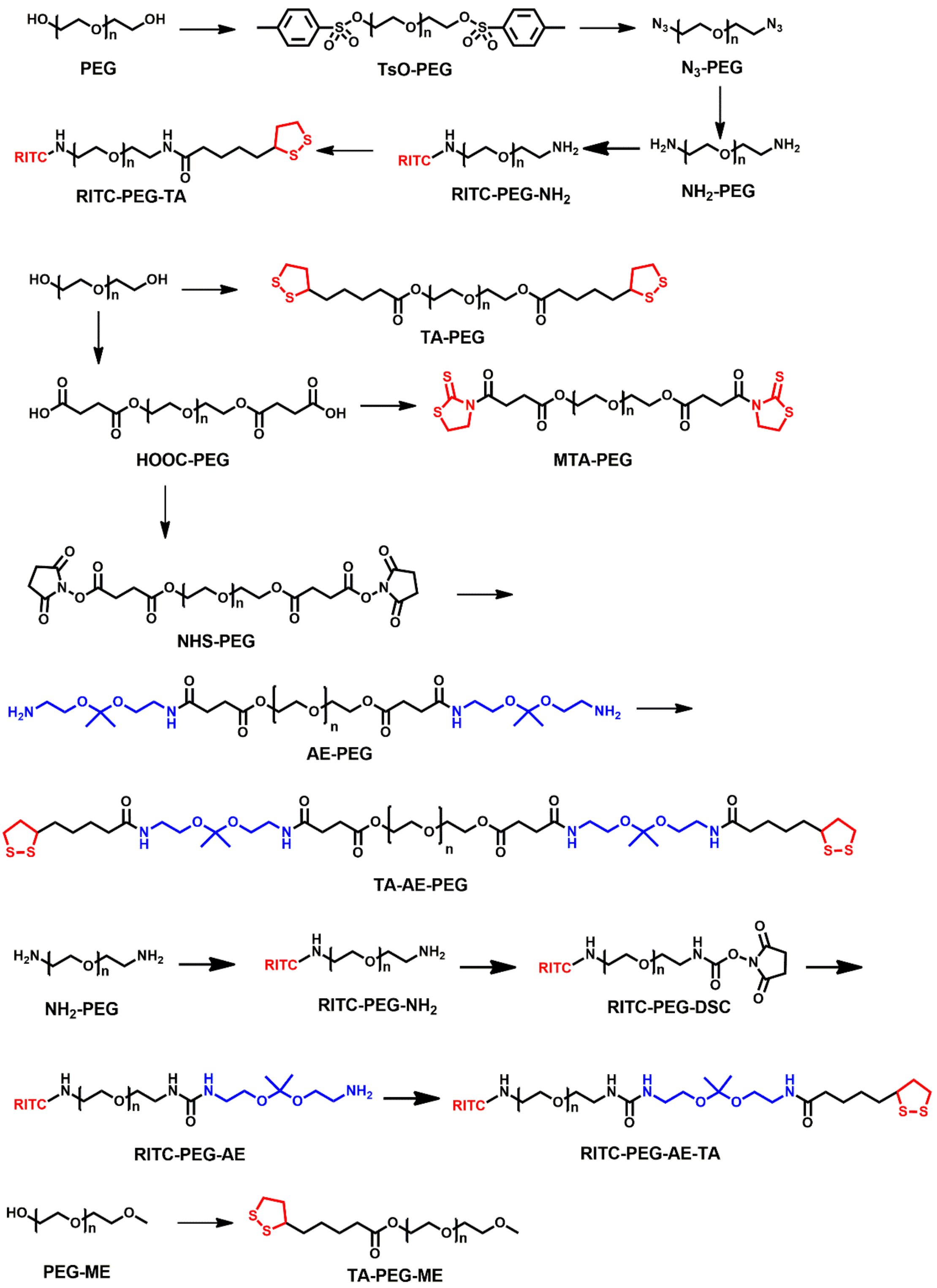

Scheme S1. General synthetic route to derivatized PEGs.

*(1) Synthesis of **TA-PEG***

Poly(ethylene glycol) (0.1 mmol), TA (0.3 mmol) and DMAP (0.06 mmol) were dissolved in dichloromethane (5 mL) in a round-bottomed flask with a magnetic stirrer. A stream of argon was bubbled through the solution for 15 minutes and the reaction vessel was cooled to 0 °C. Then, DCC (0.3 mmol, dissolved in 0.5 mL dichloromethane) was injected into the solution. The reaction was stirred at room temperature for 24 hours. The precipitate was filtered off and the residue was added dropwise into 200 mL cool diethyl ether. The TA-PEG was recovered by filtration as white solid. ^{1}H-NMR (400 MHz, $CDCl_3$): δ(p.p.m.) 4.20 (t, 4H), 3.7-3.5 (m, H of PEG main chain), 3.10 (m, 4H), 2.45 (m, 2H), 2.33 (t, 4H), 1.89 (m, 2H), 1.7-1.55 (m, 8H), 1.5-1.35 (m, 4H).

*(2) Synthesis of the **HOOC-PEG***

Poly(ethylene glycol) (0.1 mmol), DMAP (0.06 mmol) and succinic anhydride (0.4 mmol) were dissolved into 50 mL chloroform in a round-bottomed flask with a magnetic stirrer and reflux condenser. A stream of argon was bubbled through the solution for 15 minutes and the reaction vessel was heated to 60 °C. The reaction was stirred at 60 °C for 18 hours. The solvent was removed by rotary evaporation. The crude product was dissolved in 10 mL dichloromethane and then precipitated by dropwise addition into 200 mL cool diethyl ether. HOOC-PEG was collected by filtration. ^{1}H-NMR (400 MHz, $CDCl_3$): δ(p.p.m.) 4.23 (t, 4H), 3.7-3.5 (m, H of PEG main chain), 2.61 (m, 8H).

*(3) Synthesis of the **MTA-PEG***

HOOC-PEG (0.1 mmol) and MTA (1 mmol) were dissolved into 50 mL dichloromethane in a round-bottomed flask with a magnetic stirrer. A stream of argon was continuously bubbled through the solution. After 30 minute degassing with argon, DCC (0.3 mmol) and DMAP (0.06 mmol) was added into the solution. The reaction mixture was stirred under argon atmosphere. After reaction for 24 hours, the precipitate was filtered off and the residue was added dropwise into 200 mL cool diethyl ether. The MTA-PEG was recovered by filtration as light-yellow solid. ^{1}H-NMR (400 MHz, $CDCl_3$): δ(p.p.m.) 4.56 (m, 4H), 4.23 (t, 4H), 3.7-3.5 (m, H of PEG main chain), 3.3 (m, 4H), 2.71 (m, 4H).

*(4) Synthesis of the **TsO-PEG***

Poly(ethylene glycol) (1 mmol) and triethylamine (10 mmol) were dissolved in 70 mL dichloromethane in a round-bottomed flask with magnetic stirrer and the reaction vessel was cooled to 0 °C. 4-Toluenesulfonyl chloride (10 mM) was added into the solution. The reaction mixture was left at room temperature with constant stirring for 24 hours. Then 100 mL deionized water was added and stirred for 1 hour. The organic layer was rotary evaporated and followed by dissolving with 40 mL dichloromethane and then precipitated by dropwise addition into 400 mL cool diethyl ether. The precipitate was washed with 200 mL cool diethyl ether and collected by filtration. ^{1}H-NMR (400 MHz, $CDCl_3$): δ(p.p.m.) 7.75 (d, 4H), 7.30 (d, 4H), 4.12 (m, 4H), 3.7-3.5 (m, H of PEG main chain), 2.41 (s, 6H).

*(5) Synthesis of the **N_3-PEG***

TsO-PEG (0.5 mmol) and sodium azide (5 mmol) were dissolved into 10 mL DMF in a round-

bottomed flask with magnetic stirrer and reflux condenser. The reaction vessel was heated to 95 °C and stirred at 95 °C for 18 hours. After cooling down to room temperature and filtration, DMF was evaporated under vacuum. The crude product was dissolved in 10 mL dichloromethane and washed twice with 20 mL saturated NaCl aqueous solution. The dichloromethane layer was dried over sodium sulfate, and precipitated by dropwise addition into 200 mL cool diethyl ether. The product was collected by filtration. ^{1}H-NMR (400 MHz, $CDCl_3$): δ(p.p.m.) 3.7-3.5 (m, H of PEG main chain), 3.37 (t, 4H).

*(6) Synthesis of the **NH_2-PEG***

N_3-PEG (0.5 mmol) and triphenylphosphine (2.5 mmol) were dissolved into 50 mL methanol in a round-bottomed flask with magnetic stirrer and reflux condenser. The reaction vessel was heated to 65 °C and stirred at 65 °C for 12 hours. After cooling down to room temperature and filtration, methanol was evaporated under vacuum. The crude product was dissolved into 20 mL dichloromethane and added dropwise into 200 mL cool diethyl ether. The product was collected by filtration. ^{1}H-NMR (400 MHz, $CDCl_3$): δ(p.p.m.) 3.7-3.5 (m, H of PEG main chain), 2.81 (m, 4H).

*(7) Synthesis of the **RITC-PEG-NH_2***

NH_2-PEG (1 g) was dissolved into 10 mL deionized water in a 25 mL glass vial. 10 mL carbonate buffer (100 mM, pH=9) was added into the vial followed by addition of RITC solution (5 mg dissolved into 2 mL DMSO). The mixture was stirred at room temperature for 12 hours, purified by dialysis (24 hours, 4 L water replaced three times) and then lyophilized to get product. The ^{1}H-NMR showed no difference with the ^{1}H-NMR of ***NH_2-PEG*** because of the low grafting ratio of RITC.

*(8) Synthesis of the **RITC-PEG-TA***

RITC-PEG-TA was synthesized using the same procedure as synthesizing TA-PEG, with the exception that RITC-PEG-NH2 was used instead of poly(ethylene glycol). ^{1}H-NMR (400 MHz, $CDCl_3$): δ(p.p.m.) 3.7-3.5 (m, H of PEG main chain), 3.10 (m, 4H), 2.44 (m, 2H), 2.17 (m, 4H), 1.89 (m, 2H), 1.70-1.55 (m, 8H), 1.45 (m, 4H). The hydrogen atoms on the RITC were not detectable because of the low grafting ratio.

*(9) Synthesis of the **NHS-PEG***

HOOC-PEG (0.1 mmol), N-hydroxysuccinimide (NHS, 0.3 mmol) and N-(3-dimethylaminopropyl)-N′-ethylcarbodiimide hydrochloride (EDC, 0.3 mmol) were dissolved into 15 mL dichloromethane in a round-bottomed flask with magnetic stirrer. The reaction mixture was stirred at room temperature. After reaction for 12 hours, 50 mL deionized water was added and stirred for 1 hour. The organic layer was washed twice with 20 mL saturated NaCl aqueous solution. The dichloromethane layer was dried over sodium sulfate, and precipitated by dropwise addition into 200 mL cool diethyl ether. The product was collected by filtration. ^{1}H-NMR (400 MHz, $CDCl_3$): δ(p.p.m.) 4.28 (m, 8H), 3.7-3.5 (m, H of PEG main chain), 2.96 (t, 4H), 2.84 (s, 8H), 2.78 (t, 4H).

*(10) Synthesis of the **AE-PEG***

NHS-PEG (0.1 mmol), 2,2-Bis(aminoethoxy)propane (0.8 mmol) and triethylamine (0.8 mmol) were dissolved in 20 mL chloroform in a round-bottomed flask with magnetic stirrer. The reaction mixture was stirred for 24 hours at room temperature, precipitated by dropwise addition into 400 mL cool diethyl ether. AE-PEG was collected by filtration. ^{1}H-NMR (400 MHz, $CDCl_3$): 4.22 (t, 4H), 3.8-3.45 (m, H of PEG main chain), 2.91 (m, 4H), 2.68 (t, 4H), 2.47 (t, 4H), 1.35 (s, 12H).

(11) Synthesis of the ***TA-AE-PEG***

TA-AE-PEG was synthesized using the same procedure as synthesizing TA-PEG, with the exception that AE-PEG was used instead of Poly(ethylene glycol). ^{1}H-NMR (400 MHz, $CDCl_3$): 4.23 (t, 4H), 3.7-3.5 (m, H of PEG main chain), 3.13 (m, 4H), 2.70 (m, 4H), 2.45 (m, 2H), 2.20 (t, 4H), 1.90 (m, 2H), 1.68 (m, 8H), 1.47 (m, 4H), 1.34 (s, 12H).

(12) Synthesis of the ***RITC-PEG-DSC***

RITC-PEG-NH_2 (1 g), triethylamine (100 µL) and disuccinimidyl carbonate (DSC, 100 mg) were dissolved in 8 mL dichloromethane in a round-bottomed flask with a magnetic stirrer. The reaction was stirred at room temperature and left to react for 18 hours. The dichloromethane solution was added into 400 mL diethyl ether dropwise. The product was collected by filtration and vacuum dried.

(13) Synthesis of the ***RITC-PEG-AE***

RITC-PEG-DSC (1 g) triethylamine (30 µL) and 2,2-Bis(aminoethoxy)propane (33 µL) were dissolved in 6 mL dichloromethane in a round-bottomed flask with a magnetic stirrer. The reaction was stirred at room temperature and reacted for 24 hours. The dichloromethane solution was added into 300 mL diethyl ether dropwise. The product was collected by filtration and vacuum dried.

(14) Synthesis of the ***RITC-PEG-AE-TA***

RITC-PEG-AE-TA was synthesized using the same procedure as for the synthesis of TA-PEG, with the exception that RITC-PEG-AE-TA was used instead of poly(ethylene glycol).

(15) Synthesis of the ***TA-PEG-ME***

TA-PEG-ME was synthesized using the same procedure as for the synthesis of TA-PEG, with the exception that poly(ethylene glycol) methyl ether (PEG-ME) was used instead of poly(ethylene glycol) (PEG).

Preparation and characterization of caged coacervate droplets

Coacervate microdroplets were prepared by mixing 250-400 µL PDDA solution (200-350 kDa, 400 mM, pH=8) and 900 µL CM-dex solution (10-20 kDa, 370 mM, pH=8). The coacervate phase was isolated by centrifugation (6000 rpm, 3 minutes). Under vortex mixing, 10 µL of the coacervate phase, 0.2-10 µL MTA-PEG or TA-PEG solution (10 mg/mL, average Mw = 20 kDa) were sequentially added into 100 µL Au-nanoparticle solution. The aqueous mixture was vigorously mixed for 1-4 minutes to afford the caged coacervate microdroplets, which were characterized by bright-field and dark-field optical microscopy by mounting a drop of the

suspension (40-50 µL) onto a sample holder. The surface charges of the native PDDA/CM-dex coacervate microdroplets and membranized counterparts were characterized using a Zeta potential analyzer.

Transmission electron microscopy

A batch of fresh-prepared Au/TA-PEG-caged coacervate droplets was left to settle for 5 minutes. Then 10 µL solution was withdrawn from the top layer and diluted by addition of 90 µL supernatant solution. After gentle shaking, 5 µL of the diluted solution was carefully dropped onto a carbon film-loaded copper grid. The sample-loaded copper grid was left for 5 minutes followed by freeze drying to remove the water phase. The mounted sample was characterized using a FEI Tecnai 20 TEM (200 kV).

Preparation of fluorescently labelled caged coacervate droplets

RITC-labelled PEG derivatives (RITC-PEG-TA, Mw = 20k) and FITC-labelled CM-dex (FITC-CM-dex) were used to determine the localization of the coacervate components by confocal laser scanning microscopy (CLSM). A coacervate dispersion was prepared by mixing 400 µL PDDA (400 mM), 900 µL CM-dex (370 mM) and 10 µL FITC-CM-dex (2 mg/mL). 10 µL of the coacervate suspension was then mixed with 100 µL Au-nanoparticle solution. 2 µL aqueous RITC-PEG-TA (10 mg/mL) was added into the solution under vigorous mixing and the dispersion of membranized coacervate microdroplets left unstirred to allow sedimentation. The upper layer of the solution containing unreacted RITC-PEG-TA was then replaced by the same volume of native coacervate supernatant.

Influence of MTA-PEG and Au concentrations on membrane stability

The stability of the membranized coacervate droplets was determined by the degree of coalescence, which was assessed by monitoring the size of the caged coacervate microdroplets using optical microscopy. 10 µL of the coacervate droplets (prepared by mixing 900 µL CM-dex and 250 µL PDDA) was mixed with a Au nanoparticle solution and MTA-PEG (10 mg/mL) aqueous solution. The final concentration of Au nanoparticles varied from 1.38×10^{13} to 7.59×10^{13} per mL. The final concentration of MTA-PEG varied from 1.8 to 45 µM. If the diameter of the largest membranized coacervate droplets was above 200 µm, the samples were regarded as highly unstable.

Reversibility of Au/TA-PEG membrane assembly

60 µL of a freshly prepared aqueous dispersion of the membranized coacervate droplets (stabilized by Au/TA-PEG) were injected into a sample holder. The droplets were illuminated at 290-390 nm for 5 min from the top at a distance of *ca*. 1 cm. The aqueous mixture was then withdrawn, added to a centrifuge tube, and vigorously sheared for 1-2 minutes by using a pipette. The aqueous mixture was then injected into a glass holder to monitor reassembly of the Au/TA-PEG membrane. The sample was then illuminated again for 5 minutes and further characterized.

Fluorescence recovery after photobleaching (FRAP)

Fluorescent membranized coacervate microdroplets were used to perform the FRAP measurements on a Leica SP5-II confocal laser scanning microscope attached to a Leica DMI 6000 inverted fluorescence microscope. A 63x oil objective lens was used for the FRAP characterization. RITC-PEG-TA was excited with a laser at 561 nm. The emission signals were collected in the range of 571-690 nm. An image was taken before photobleaching using a 561 nm laser at 100% intensity for 10 frames (1.293 s/frame). The fluorescence recovery was recorded for 10 minutes (30 s/frame). FITC-CM-dex was excited with a laser at 488 nm. The emission signals were collected in the range of 498-550 nm. An image was taken before photobleaching using a 488 nm laser at 100% intensity for 2 frames (1.293 s/frame). The florescence recovery was recorded for 1 minutes (5 s/frame). The FRAP analysis was carried out by using ImageJ software with a "Creat spectrum jru v1" plugin.

Number of PEG moles per Au nanoparticle

Typically, the caged coacervate micro-droplets were prepared using a PEG-derivative concentration of 3.6-27 µM [**n**(PEG)]. The concentration of Au nanoparticles was typically 3.45 x 10^{13} mL^{-1}. The number of PEG molecules was given by **n**(PEG) x N_A = 2.17-16.3 x 10^{15} mL^{-1} (N_A = 6.022 x 10^{23} mol^{-1}), indicating that on average 63 to 472 PEG molecules were attached to each Au nanoparticle.

Permeability of the Au/TA-PEG membrane

1 µL of calcein, RITC, FITC-labelled CM-dextrin, RITC-labelled PDDA, FITC-labelled Bovine serum albumin and RITC-labelled amylase (1 mg/mL in aqueous solution) were added into 30 µL freshly prepared Au/TA-PEG20k-membranized coacervate microdroplet suspension, respectively. The aqueous mixture was stirred using a pipette for 10 seconds. The permeability of the fluorescent-labelled molecules was characterized by confocal laser scanning microscopy (CLSM).

Light-mediated uptake of ZIF8 particles in caged coacervate droplets

Microparticles of a zeolitic imidazolate framework (ZIF8) with fluorescently labelled guest bovine serum albumin molecules were prepared as follows. An aqueous solution (4.4 mL) containing 2 mg RITC-labelled BSA and 0.41 g 2-methylimidazole was mixed with aqueous $Zn(NO_3)_2$ (0.4 mL, 37 mg) under stirring at room temperature. After 30 min, the product (RITC-BSA@ZIF8) was collected by centrifuging at 4 000 rpm for 6 min and washed twice with deionized water. The prepared microparticles (mean size = 0.5 µm) were then dispersed in 5 mL deionized water.

10 µL of a freshly prepared aqueous dispersion of RITC-BSA@ZIF8 particles were gently added to a region of the freshly prepared membranized coacervate microdroplet suspension and stirred using a pipette for 10 seconds. 60 µL of the solution was then injected into a pegylated glass sample holder and left for several minutes to allow the caged coacervate droplets to settle. The droplets were illuminated for 6 min from the top of the sample holder at a distance of approximately 1 cm using light with a wavelength of 290-390 nm. CLSM images were recorded before and after light illumination.

Light-mediated capture of polyoxometalate coacervate vesicles (PCVs) by caged

coacervate droplets

PCVs were prepared as follows. PDDA (12.5 mM, 400 µL, pH 6.5), ATP (2.5 mM, 400 µL, pH 6.5) and RITC-labelled amylase (2 mg/mL, 10 µL) solutions were added to a 1.75 mL vial under sonication to form PDDA/ATP coacervate microdroplets. PTA (20 mM, 100 µL) was then quickly injected, and the solution sonicated for another one minute to produce stable PCVs. The PCVs were centrifuged and the supernatant was replaced with the same volume of deionized water. The washing process was repeated two times. The washed PCVs were dispersed in 100 µL deionized water.

An aqueous mixture containing 10 µL PCV and 50 µL of Au/TA-PEG-caged coacervate droplet dispersions was injected into a pegylated glass sample holder and the microscale objects allowed to sediment for 5 minutes. The mixed dispersion was then illuminated for 10 min from the top of the sample holder at a distance of approximately 1 cm using light with a wavelength of 290-390 nm, and monitored *in situ* by optical microscopy.

Preparation of silica colloidosomes

An aqueous PBS buffer solution (pH=7) containing 20 mg/mL RITC-labelled amylase was dispersed into 3 mL toluene containing 35 mg of partially hydrophobic SiO_2 nanoparticles. The aqueous mixture was sonicated for 3-4 minutes to produce a water-in-oil Pickering emulsion. Immediately after emulsification, 10 µL of tetramethoxysilane was added and the liquid mixture rotated for 24 hours. The crosslinked Pickering emulsion (colloidosomes) was transferred into an aqueous solution by continuous washing with 70% ethanol : water, 50% ethanol : water followed by pure water. The colloidosomes were dispersed in 1.0 mL water.

Preparation of PEG-tagged colloidosomes

An aqueous PBS buffer solution (pH=7) containing 20 mg/mL FITC-labelled amylase was dispersed into 3 mL toluene containing 35 mg of partially hydrophobic SiO_2 nanoparticles. The aqueous mixture was sonicated for 3-4 minutes to produce a water-in-oil Pickering emulsion. Immediately after emulsification, 10 µL tetramethoxysilane was added and the liquid mixture rotated for 8 hours. 10 µL of 3-[methoxy(polyethyleneoxy)propyl]trimethoxysilane was then added and the liquid mixture rotated for 16 hours. The crosslinked Pickering emulsion (PEG-tagged colloidosomes) was transferred into aqueous solution by continuous washing with 70% ethanol : water, 50% ethanol : water followed by pure water. The PEG-tagged colloidosomes were dispersed into 1.0 mL water.

Light-mediated capture of colloidosomes

10 µL of a freshly prepared aqueous dispersion of RITC-amylase-containing colloidosomes were mixed with 90 µL of an aqueous suspension of caged coacervate droplets and stirred using a pipette for 10 seconds. 60 µL of the mixture was injected into a sample holder and illuminated with light (290-390 nm) from the top of the sample holder at a distance of *ca*. 1cm. After 10 minutes of light illumination, the sample was stirred using a pipette for 1 min. The sample was characterized by CLSM before and after light illumination.

NMR spectroscopy studies of TA-AE-PEG hydrolysis

Hydrolysis of TA-AE-PEG as a function of pH (from pH 7.0 to pH 5.3) was investigated. 10-15 mg of TA-AE-PEG was dissolved in deuterated phosphate buffer solution (PBS, 100 mM, 0.5 mL) prepared at different pH values. The NMR spectra were acquired in a one-hour period with a 15-minute interval. The acquired spectra were analysed by integrating the peaks at 1.33 ppm (ketal) and 2.16 ppm (acetone).

Chemical-mediated capture of PCVs

Caged coacervate droplets were prepared by using Au/TA-AE-PEG6k nanoparticles as a nanoparticle surfactant. 50 µL of the aqueous dispersion was mixed with 10 µL of FITC-labelled GOx aqueous solution (final concentration, 0.2 mg/mL). The FITC-labelled GOx preferentially partitioned into the caged coacervate droplets by transport through the nanoporous membrane. The aqueous suspension of caged coacervate droplets was then mixed with 10 µL of RITC-labelled PCVs. Addition of glucose (final concentration, 10 mM) triggered the chemical-mediated capture of the PCVs. The protocells were characterized before and 60 minutes after the addition of glucose.

FACS analysis

FACS characterization was carried out using a Canto II flow cytometer operated at a low pressure with a 100 µm sorting nozzle. At least 10^6 particles were characterized to determine the 2D dot plots of the FSC and SSC light. The fluorescence signals of the individual particles were characterized by 488 and 565 nm lasers. Sample characterizations for FACS were as follows: (**i**) an aqueous mixture containing 20 µL of RITC-labelled PCVs and 280 µL water was characterized to determine the FACS signal for an individual population of PCVs ; (**ii**) 300 µL aqueous suspension containing the FITC-GOx-loaded caged coacervate droplets was characterized to determine the FACS signal of an individual population of caged coacervate micro-droplets; (**iii**) for chemical-mediated uncaging, an aqueous suspension containing 20 µL RITC-labelled PCVs and 300 µL GOx-loaded (0.2 mg/mL) caged coacervate droplets were characterized by FACS before and 1 hour after the addition of 10 mM glucose. The aqueous suspension was pipetted for 10 seconds before FACS characterization. Time series of counting were obtained and FlowJo 10.6 software was used for all the data analysis.

Chemical-mediated protocell sorting

An aqueous suspension containing 10 µL of a dispersion of PEG-tagged colloidosomes (FITC-labelled) and 10 µL of a PCV (RITC labelled) dispersion was mixed with 100 µL GOx-loaded (0.2 mg/mL) caged coacervate droplets. 10 mM glucose was added into the aqueous suspension and the system left to react for 1 hour. The aqueous suspension was pipetted for 1 minute and then left tp settle down for 3 minutes. 50 µL aqueous suspension was gently withdrawn from the top layer of the aqueous suspension, and diluted to 300 µL by addition of deionized water for FACS characterization. An aqueous suspension containing 10 µL of a dispersion of PEG-tagged colloidosomes (FITC-labelled) and 10 µL of a PCVs (RITC labelled) dispersion was diluted by addition of 280 µL water for FACS characterization. The aqueous suspension was left to react for 1 hour after the addition of glucose. Samples were also characterized by CLSM.

Preparation of fluorescently labelled enzymes

Enzyme solutions (4 mg/mL) were prepared by dissolving the enzyme powder in 10 mL of 100 mM carbonate buffer at pH=9. 200 µL of a DMSO solution of FITC or RITC (2 mg/mL) was added dropwise and the reaction was magnetically stirred for 12 hours at 4 °C, purified by dialysis (12 hours, 2.4 L water replaced three times) and then lyophilized. The prepared fluorescently labelled enzymes were stored at -20 °C under an argon atmosphere.

Pegylation of coverslips and preparation of the sample holder

Coverslips were pegylated to reduce coacervate wetting. Firstly, the coverslips were rinsed by ethanol and dried with compressed air. Then, the coverslips were incubated at room temperature for overnight in a toluene solution containing 1 vol% 3-[methoxy(polyethyleneoxy)propyl]trimethoxysilane. Finally, the treated coverslips were rinsed with ethanol, dried with compressed air, and stored in a desiccator for further use. Sample holders used for sample observation were prepared by mounting a pegylated coverslip onto a commercial glass slide with a home-made aperture (hole diameter, 8 mm). The treated coverslip was bonded to the glass slide via UV-curing glue to seal one side of the aperture. The sample was injected into the aperture and mounted onto the treated coverslip for observation. The role of the drilled glass slide was to confine the sample solution on the treated coverslip and avoid water evaporation.

Light sources

We used a MAX-303 Xenon Light Source (300W) equipped with UV and VIS modules. 290-390 nm light was generated by the UV module without a bandpass filter. 500-520 nm light or 480-500 nm light were generated by the VIS module together with the corresponding bandpass light filter. A quartz light guide (inner diameter, 5mm; length, 1m) was connected to the light source and used for light transmission. The light intensity of the UV module was measured by an accumulated UV power meter UIT-150 S365 Uship and was 1699 mW/mm^2. The distance between the light source and detector was 10 mm. The whole illumination area was a circle with diameter of *ca*. 9mm. For more information of the light source, see https://www.gmp.ch/htmlarea/pdf/asahi_pdf/max303techinfo.pdf

Part 2. Movie legends

Movie 1.

Optical microscopy video showing light-mediated capture of PCVs in two Au/TA-PEG caged coacervate droplets present amongst a dense population of PCVs (smaller objects). Interaction with the PCVs results in translocation into the caged coacervate droplets. Movie is shown at x15 of real-time speed.

Part 3. Supplementary Figures

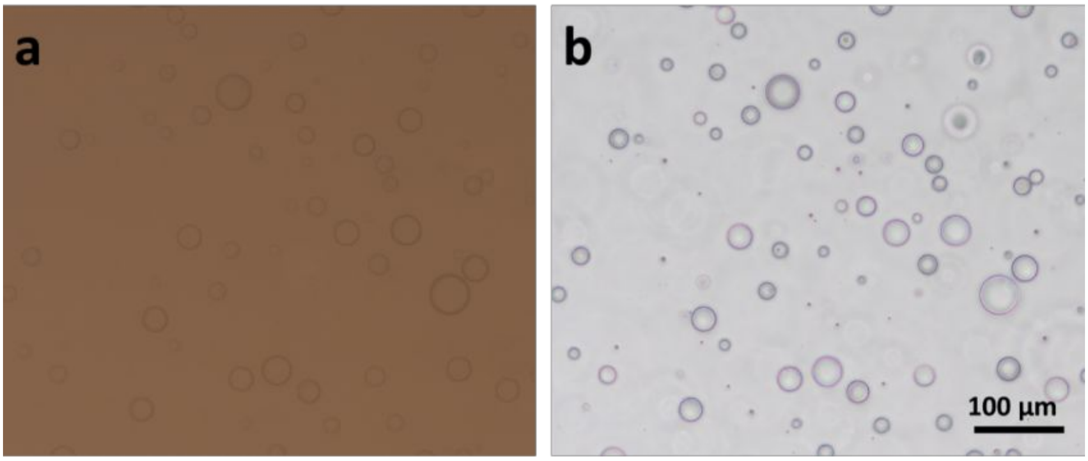

Figure S1. (a) Bright field and (b) phase field microscopy images showing PDDA/CM-dex coacervate microdroplets. The coacervate microdroplets were prepared by mixing 900 µL CM-dex (20 mM) and 400 µL PDDA (20 mM). Scale bar, 100 µm for both images.

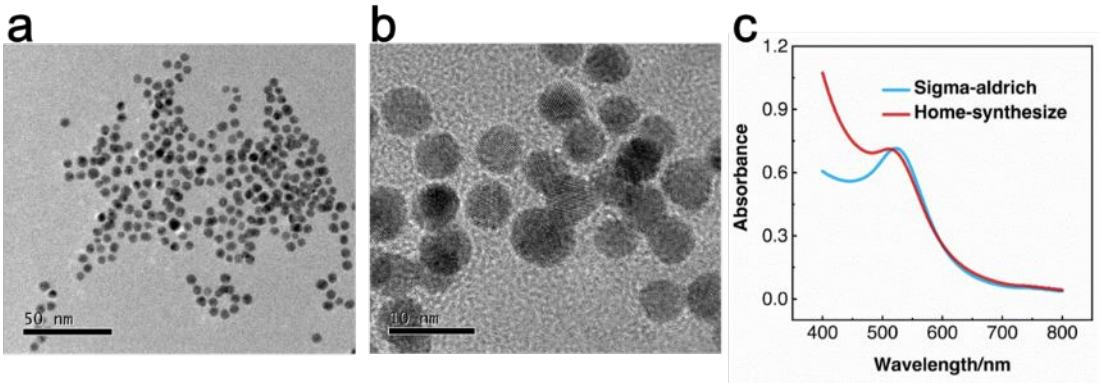

Figure S2. (a,b) Transmission electron microscopy (TEM) images showing tannic acid-coated Au nanoparticles. (c) Corresponding UV-vis spectrum showing characteristic absorbance at around 520 nm. The commercial Au-nanoparticle solution (Sigma-Aldrich) was diluted to 69% of the original concentration. Scale bars are 50 nm (a) and 100 nm (b).

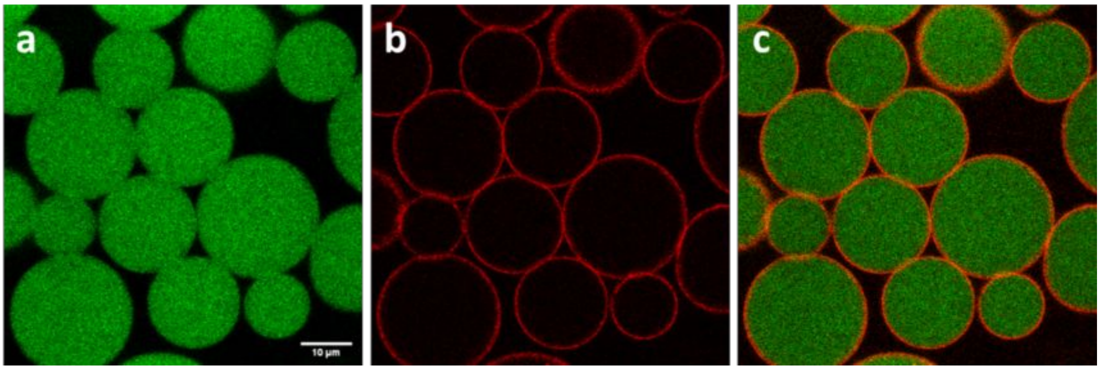

Figure S3. CLSM images of Au/TA-PEG nanoparticle-caged PDDA/CM-dex coacervate droplets. (a) Green fluorescence filtered image (FITC-labelled CM-dex), (b) red fluorescence filtered image (RITC-PEG-TA); (c) green/red superimposed image. Scale bar, 10 µm for all images.

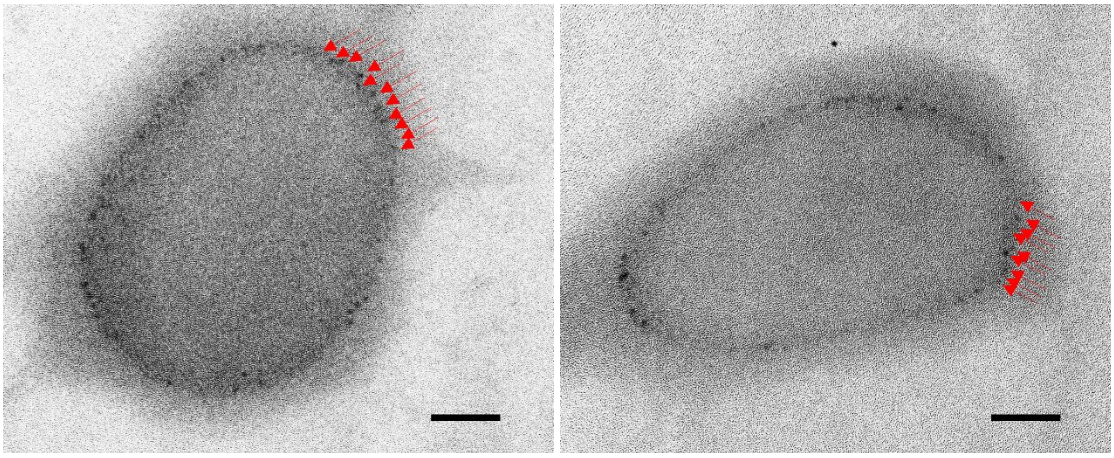

Figure S4. Transmission electron microscopy (TEM) images showing location of Au nanoparticles at the surface of caged coacervate droplets. Red arrows indicate various locations of the Au nanoparticles. Scale bars, 50 nm.

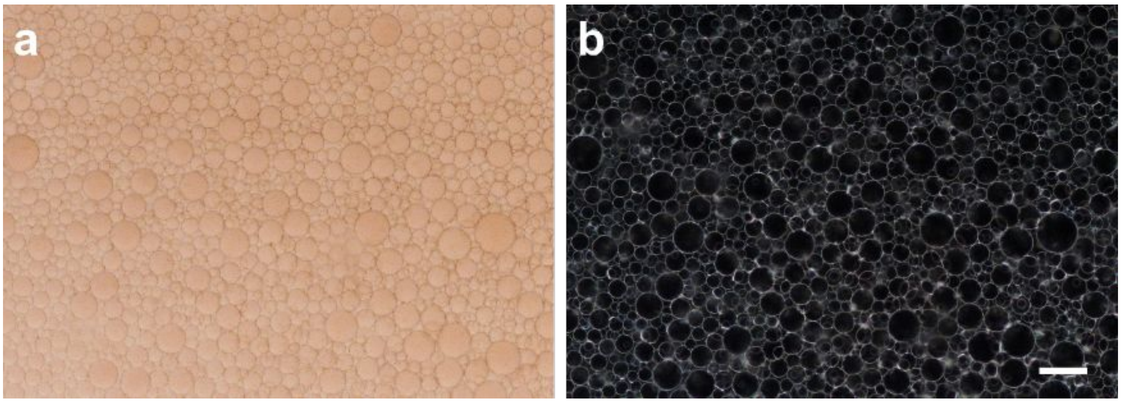

Figure S5. (a) Bright field and (b) dark field microscopy images showing Au/MTA-PEG-caged coacervate micro-droplets. Scale bar, 100 µm.

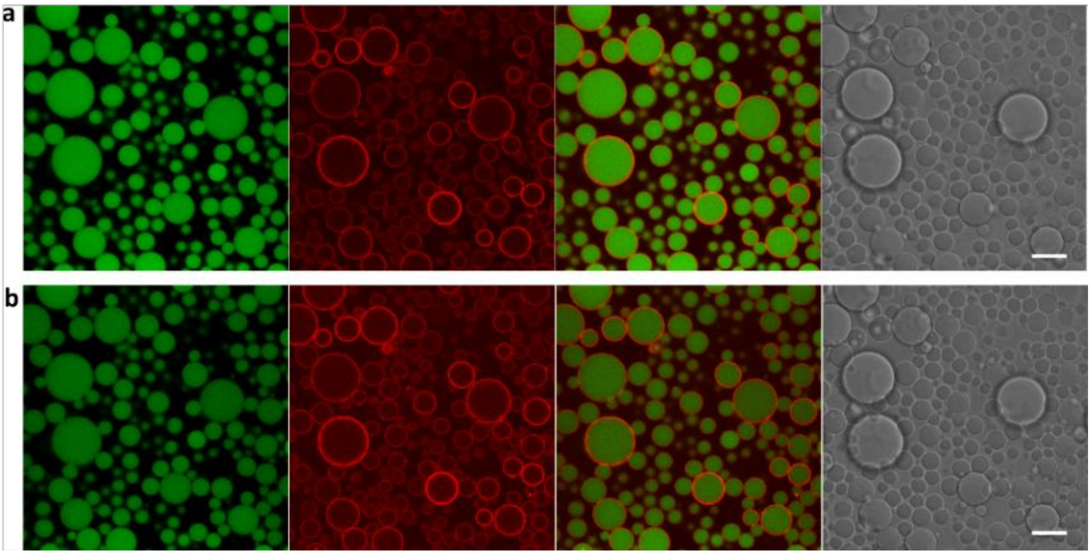

Figure S6. (a) CLSM images of green (left) and red (left middle) filtered images, green/red superimposed images (right middle) and bright-field images (right) showing Au/TA-PEG-ME nanoparticle-caged coacervate micro-droplets. Green fluorescence, FITC-labelled CM-dex. Red fluorescence, RITC-labelled TA-PEG. (b) CLSM images of the same Au/TA-PEG-ME nanoparticle-caged coacervate micro-droplets as in (a) but after being left in the characterization chamber for 10 minutes. The Au/TA-PEG-ME-caged coacervate micro-droplets are stable under the light source. Similar experiments showed that the Au/TA-PEG nanoparticles were also stable under the same conditions. Scale bars, 20 µm.

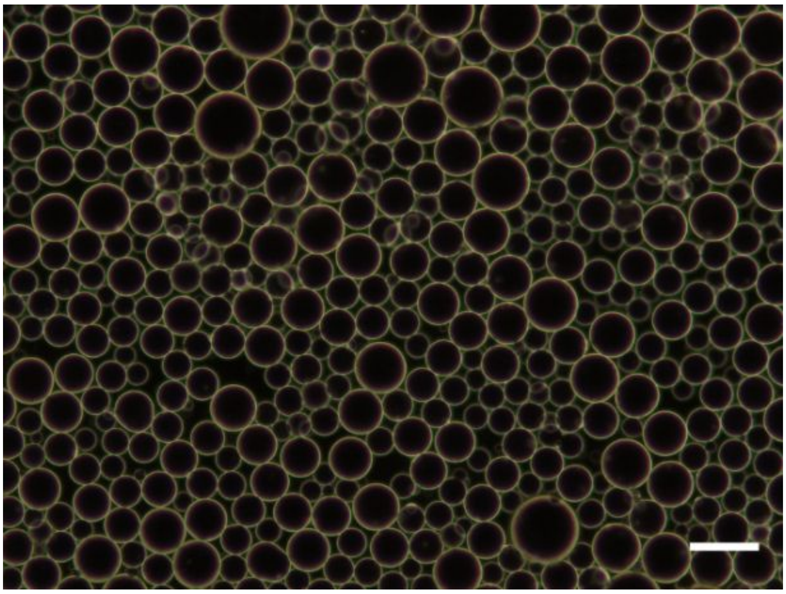

Figure S7. Dark field microscopy images showing Au/TA-AE-PEG-caged coacervate micro-droplets. After preparation, the sample was collected from the top layer of the aqueous solution. Scale bar, 100 µm.

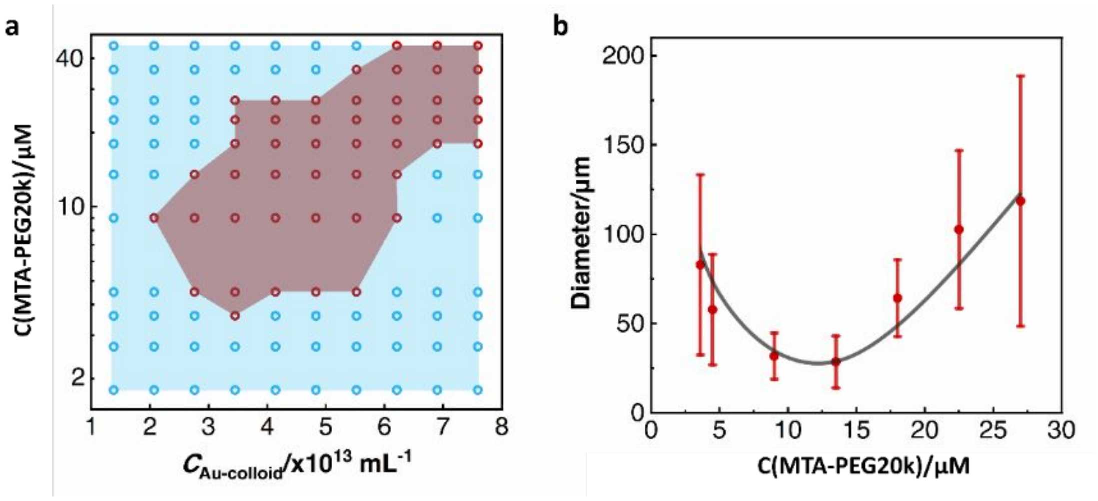

Figure S8. (**a**) Phase plot showing the influence of MTA-PEG and Au-nanoparticle concentrations on the stability of the prepared nanoparticle-caged coacervate droplets. Red circles/red background indicate stable microstructures while blue circles/blue background indicate non-stable dispersions. (b) Plot of diameter of caged coacervate droplets against MTA-PEG concentrations at a fixed Au-nanoparticle concentration of 3.5 x 10^{14} particles per mL. Error bars represents the standard deviation (n=100). A minimum in the droplet diameter is observed at an optimal MTA-PEG concentration of approximately 12.5 μM. This is because at concentrations below 12.5 μM, increasing the MTA-PEG concentration generates more Janus-like nanoparticle surfactants, which enables a higher surface area to be stabilized by the surface-active properties of Au/MTA-PEG. This is similar to a conventional Pickering emulsion system. However, at concentrations above 12.5 μM, an increasing proportion of the Au nanoparticles become completely covered in the polymer, which hampers their surface activity. Thus, the diameter of the caged droplets increases and the system becomes more polydisperse.

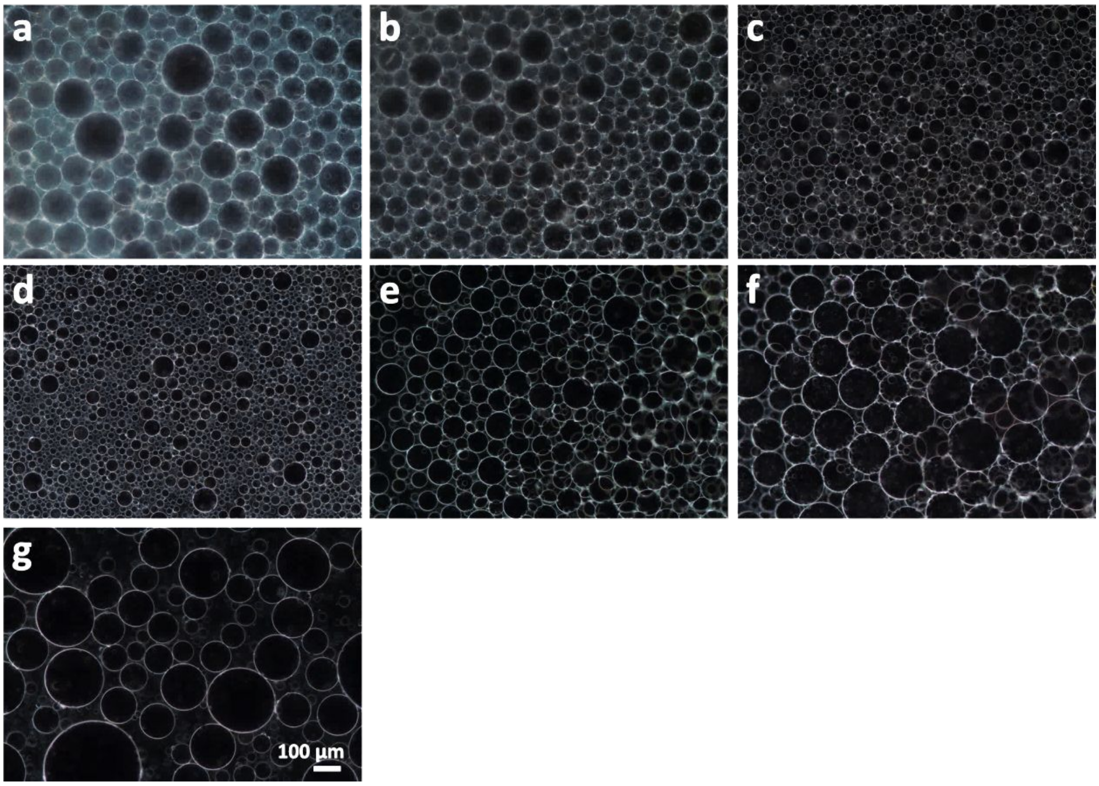

Figure S9. Dark-field optical images showing caged coacervate droplets prepared by adding tannic acid-capped Au nanoparticles followed by different concentrations of MTA-PEG (Mw = 20k) into an aqueous mixture (total volume 110 µL) containing 1.6×10^{-5} M PDDA, 3.33×10^{-5} M CM-dex and 3.45×10^{13} mL^{-1} Au nanoparticle. MTA-PEG concentrations: (a) 3.6 µM; (b) 4.5 µM; (c) 9 µM; (d) 13.5 µM; (e) 18 µM; (f) 22.5 µM; (g) 27 µM. Scale bar is 100 µm.

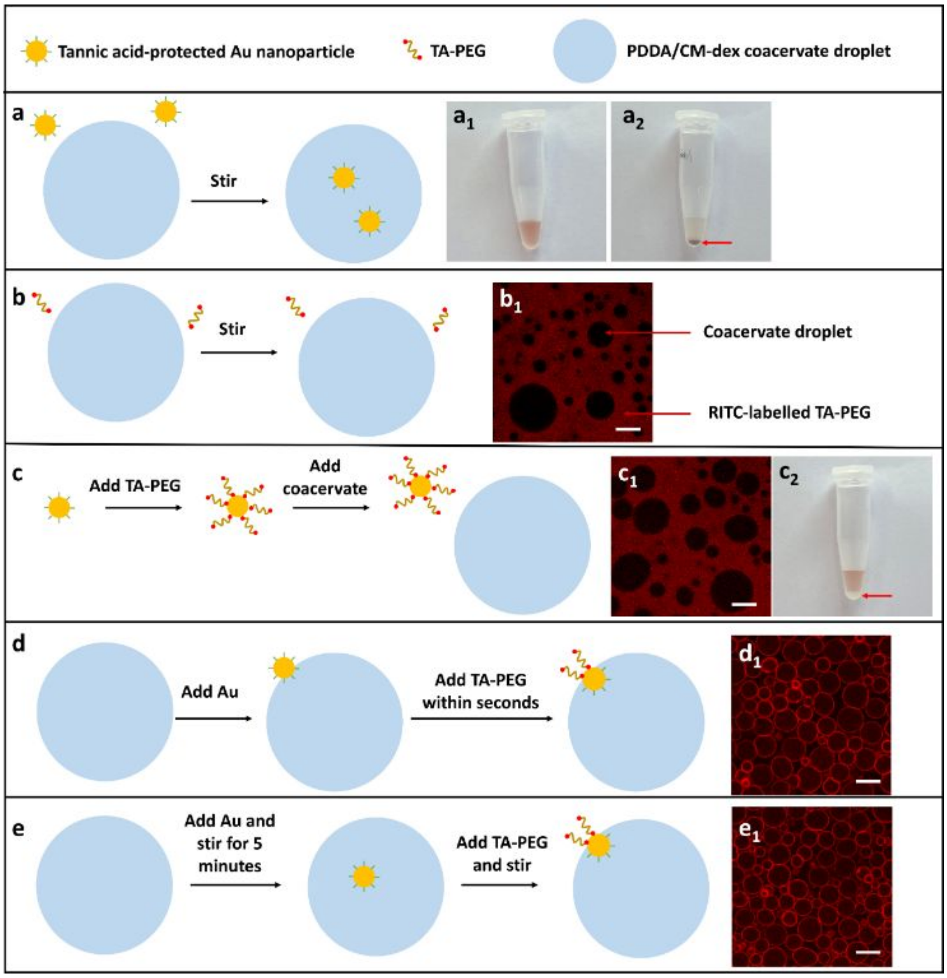

Figure S10. Self-assembly mechanism of the caged coacervate micro-droplet formation. (a) Addition of coacervate droplets into an aqueous dispersion of tannic acid-protected Au nanoparticles leads to the spontaneous accumulation of the Au nanoparticles in the coacervate droplets, indicating that the tannic acid-coated Au nanoparticles strongly interact with the coacervate droplets. Photographs show an aqueous dispersion of Au nanoparticles before (a_1) and after (a_2) the addition of the coacervate phase. The Au nanoparticles are sequestered into the coacervate phase (red arrow in (a_2)). (b) Addition of coacervate droplets into an aqueous solution of RITC-labelled TA-PEG showing that TA-PEG is excluded from the coacervate phase and remains in the continuous aqueous phase. (b_1) CLSM image showing red fluorescence from RITC-PEG-TA only in the external continuous phase of a suspension of PDDA/CM-dex coacervate droplets (black circles, no fluorescence). (c) Addition of TA-PEG into a tannic acid-protected Au nanoparticle solution leads to ligand exchange on the Au nanoparticles. Because of this, addition of coacervate droplets does not result in uptake and the nanoparticles remain in the continuous phase, as confirmed by the CLSM image (c_1) and optical image (c_2). The red arrow delineates the coacervate phase. (d) Addition of tannic acid-protected Au nanoparticles to a coacervate droplet suspension followed within a few seconds by addition of RITC-labelled TA-PEG leads to spontaneously membranization of the coacervate droplets, implying that interfacial assembly occurs due to an asymmetric ligand coverage associated with the *in situ* formation of amphiphilic nanoparticles. (e) Addition of tannic acid-protected Au nanoparticles to a coacervate droplet suspension followed by stirring for 5 minutes leads to the accumulation of the Au nanoparticles in the coacervate droplets. If RITC-labelled TA-PEG is then added to the continuous phase along with vigorous stirring, membranization of the coacervate droplets occurs, consistent with an asymmetric distribution of ligands. (e_1) CLSM image shows the membranized coacervate droplets. Scale bars are 50 μm.

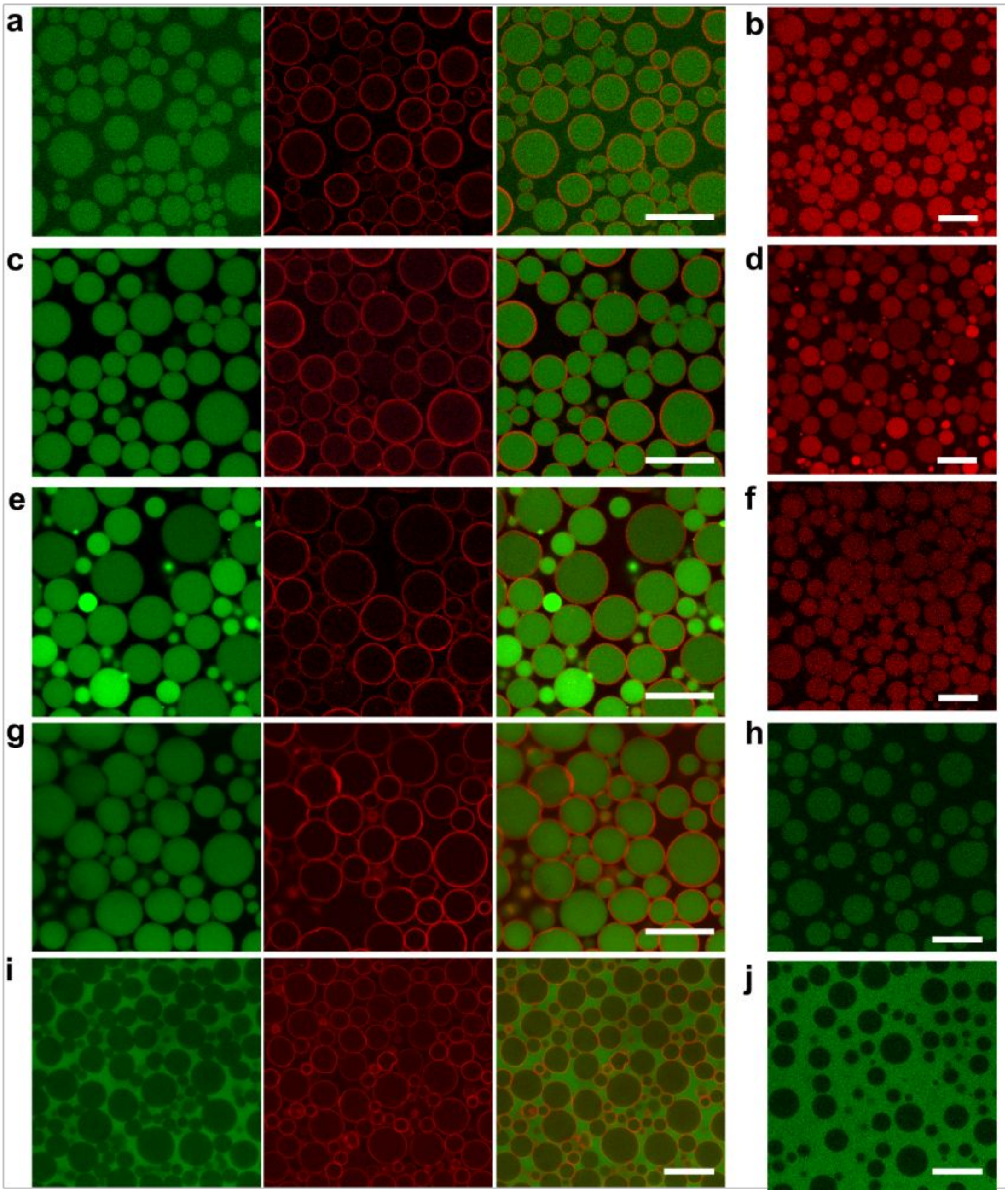

Figure S11. (a,c,e) CLSM images of green (left) red (middle) and green/red superimposed (right) channels show that the caged coacervate micro-droplets exhibit high permeability to calcein (a), FITC-labelled albumin bovine serum (c) and FITC-labelled CM-dextrin (e). Red fluorescence, RITC-labelled TA-PEG. (b,d,f) CLSM images show that the caged coacervate micro-droplets exhibit high permeability to RITC (b), RITC-labelled PDDA (d) and RITC-labelled amylase (f). All the fluorescently labelled molecules were added into the water phase and taken up and concentrated by the caged coacervate micro-droplets. (g) CLSM images of green (left) red (middle) and green/red superimposed (right) channels show that the caged coacervate micro-droplets exhibit high permeability to FITC-labelled dextran (Mw=4k). (h) CLSM images show that the uncoated coacervate micro-droplets accumulate FITC-labelled dextran (Mw=4k). (i) CLSM images of green (left) red (middle) and green/red superimposed (right) channels show that the caged coacervate micro-droplets do not take up FITC-labelled dextran (Mw=40k). (j) CLSM images show that the uncoated coacervate micro-droplets also do not accumulate FITC-labelled dextran (Mw=40k).

Scale bars, 50 µm.

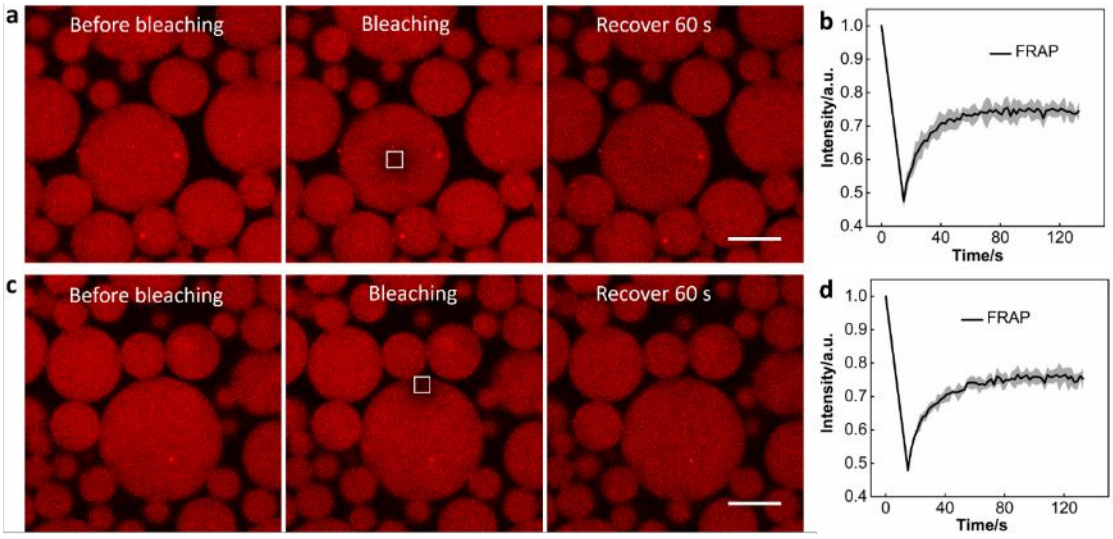

Figure S12. Fluorescence recovery after photobleaching (FRAP) experiments. (a) Time series of CLSM images of Au/TA-PEG-caged coacervate droplets (red fluorescence, RITC-labelled PDDA) and (b) corresponding changes of fluorescence intensity. The photobleached area is delineated by the white rectangle and is located at the centre of the droplet. (c) Time series of CLSM images of Au/TA-PEG-caged coacervate droplets (red fluorescence, RITC-labelled PDDA) and (d) corresponding changes of fluorescence intensity. The photobleached area is delineated by the white rectangle and is located at the boundary of the droplet. The half-life periods for fluorescent recovery are 23±1 s close to membrane and 22.7±1.2 s in the core, indicative of a liquid-like phase. Scale bars are 20 µm for all images. Error bars represent the standard deviation (n=3).

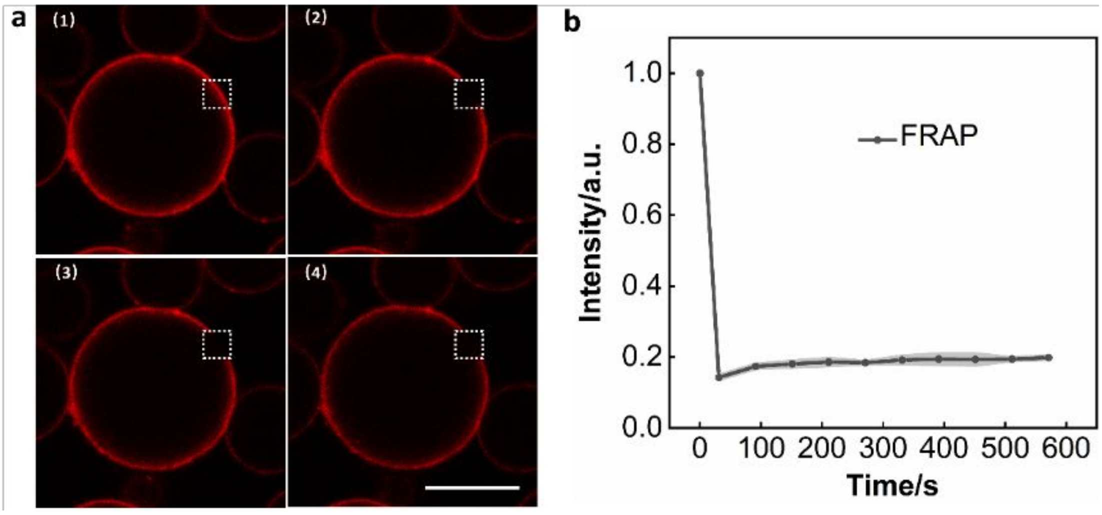

Figure S13. (a)Time series of CLSM images of Au/TA-PEG-ME nanoparticle-caged coacervate micro-droplets recorded before (**1**), 30s after bleaching (**2**), 5 minutes after recovery (**3**) and 10 minutes after recovery (**4**). The photobleached area is delineated by white rectangles and corresponds to the jammed nanoparticle membrane. Red fluorescence, RITC-labelled TA-PEG. As observed for bidentate Au/TA-PEG, a solid-like membrane is also produced using the monodentate ME ligand. Scale bar is 20 µm. (b) Plots of changes in fluorescence intensity for delineated area shown in (a). Error bars represent the standard deviation (n=3).

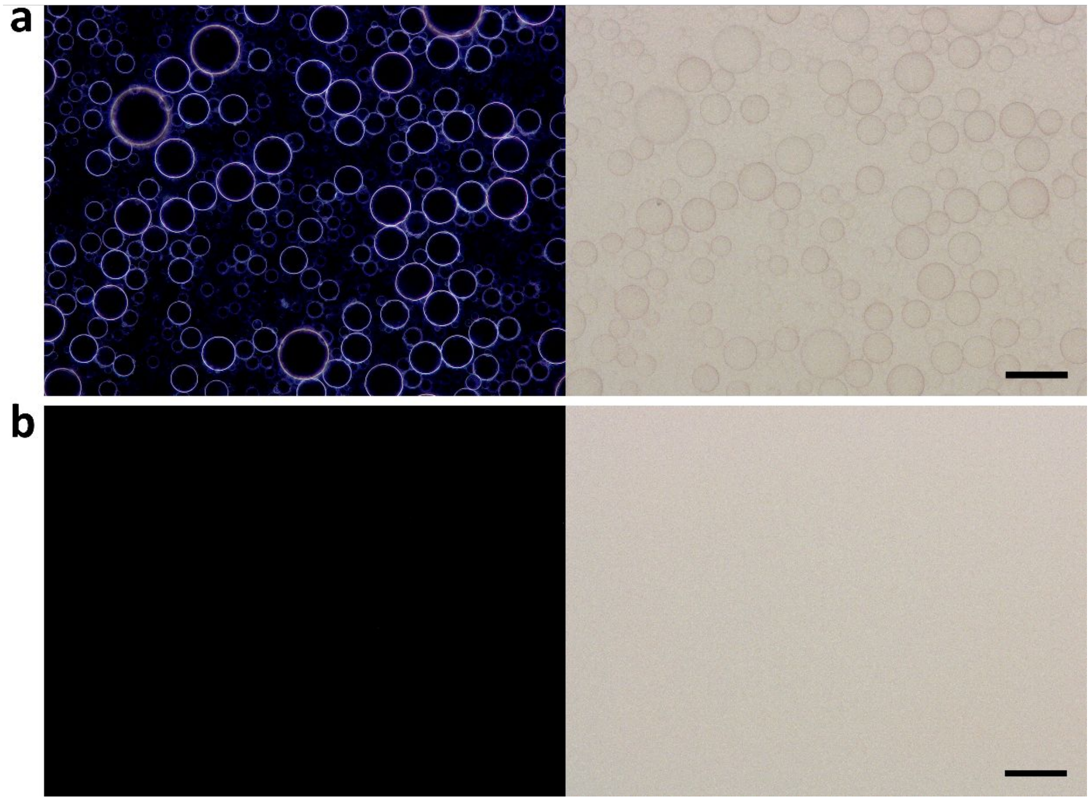

Figure S14. (a) Dark field (left) and bright field (right) microscopy images showing Au/TA-PEG-caged coacervate micro-droplets. (b) Dark field (left) and bright field (right) microscopy images showing Au/TA-PEG-caged coacervate micro-droplets after dissolving the coacervate interior by slow addition of distilled water. The nanoparticle membrane is completely disassembled, suggesting that the nanoparticles were not crosslinked by the bidentate ligand. Scale bars, 50 μm.

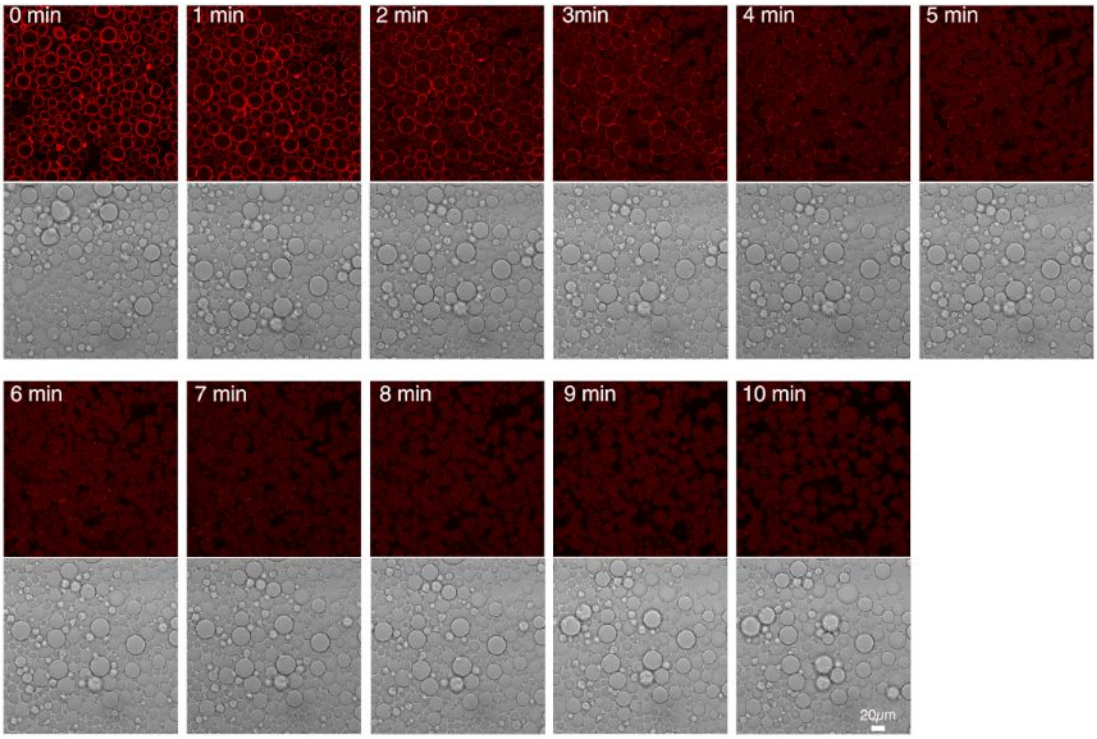

Figure S15. Time series of CLSM images (top: fluorescent field; bottom: bright field) of Au/RITC-TA-PEG nanoparticle-caged coacervate droplets after continuous light illumination. Progressive decreases in membrane red fluorescence and increased red fluorescence in the coacervate interior are observed as the jammed Au nanoparticles unlock. Scale bar, 20 µm.

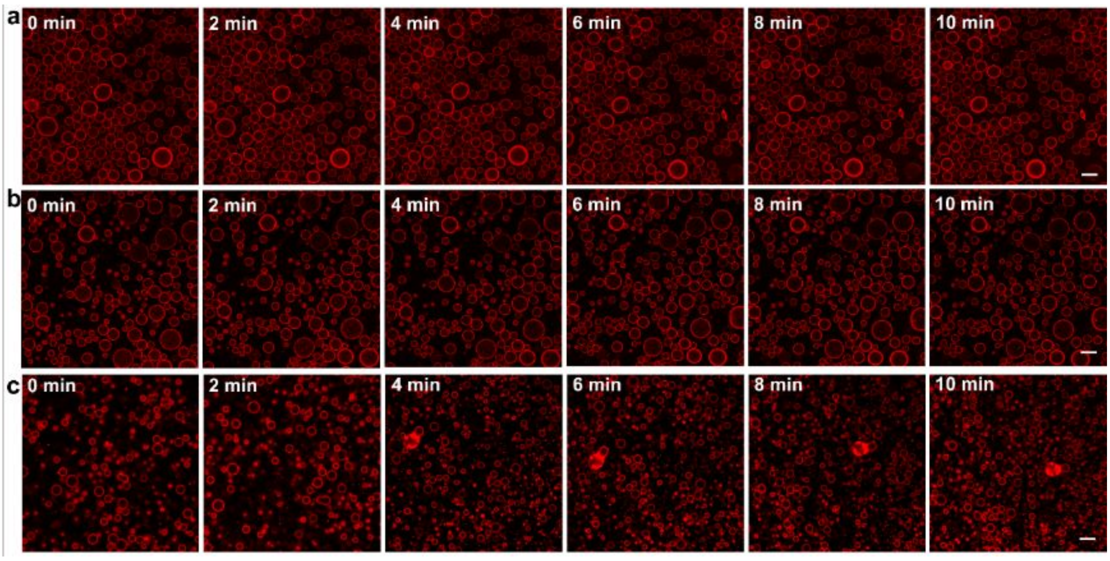

Figure S16. (a-b) Time series of CLSM images of Au/RITC-TA-PEG nanoparticle-caged coacervate droplets after continuous light (a, 480-500nm; b, 500-520nm) illumination. No obvious membrane disassembly was observed within 10 minutes. (c) Time series of CLSM images of RITC-labelled PCVs under light (290-390nm) illumination. No photobleaching effect was observed during the ten-minute period. Scale bars, 20 µm.

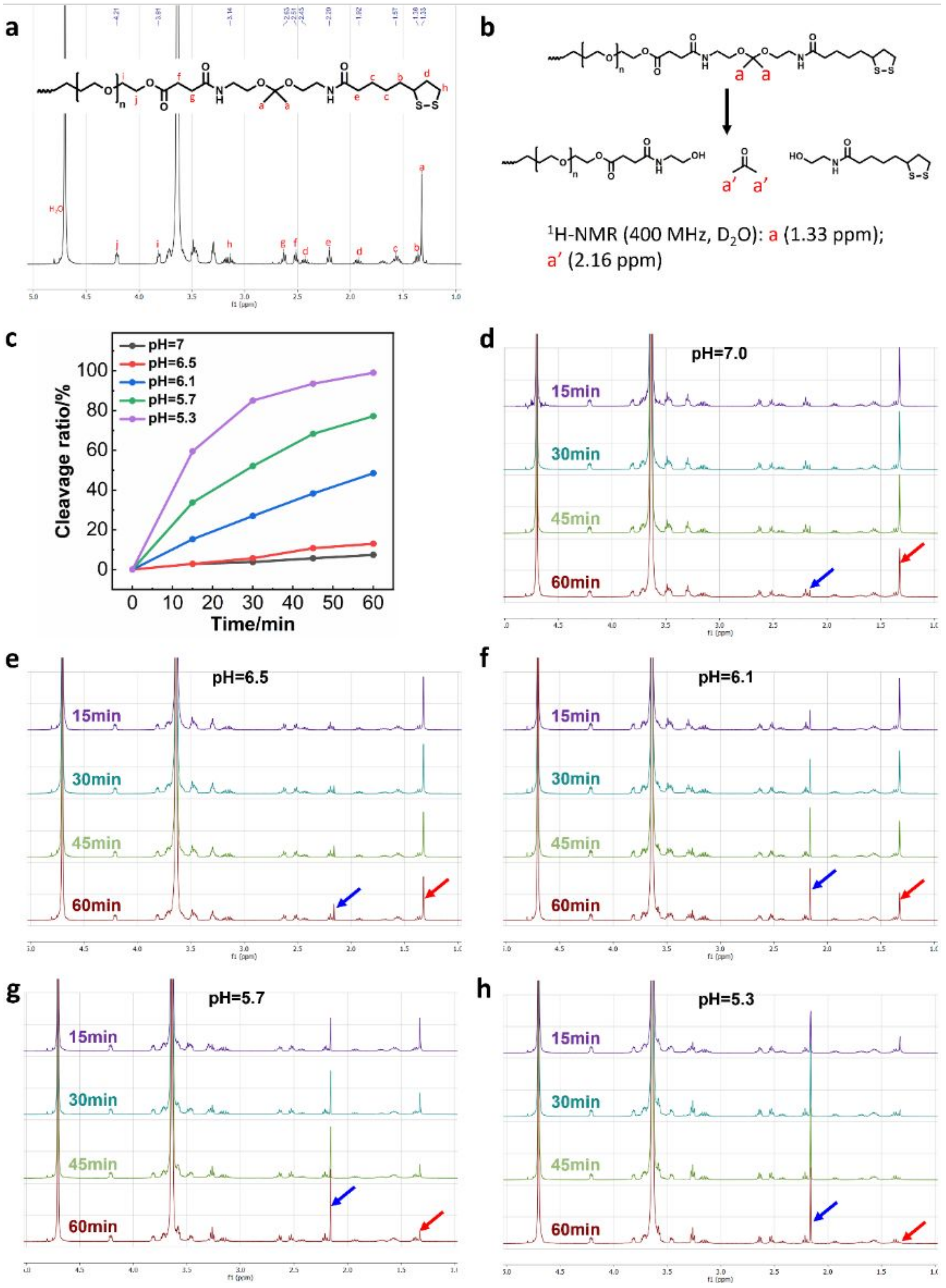

Figure S17. (a) ^{1}H-NMR (400 MHz D_2O, pH=9) spectra of TA-AE-PEG. (b) Scheme shows the H^+-mediated cleavage of TA-AE-PEG. The ketal peak at 1.33 ppm and acetone peak at 2.16 ppm were used to determine the cleavage ratio. (c) Plot shows the time-dependent cleavage ratio of TA-AE-PEG when incubated into deuterated phosphate buffer solution (pH values between 7.0 to 5.3). (d-h) Time-series ^{1}H-NMR (400 MHz D_2O) spectra of TA-AE-PEG when incubated into deuterated phosphate buffer solution (100 mM). The pH was varied from 7.0 to 5.3. The red and blue arrows indicate characteristic peaks of the ketal and acetone, respectively.

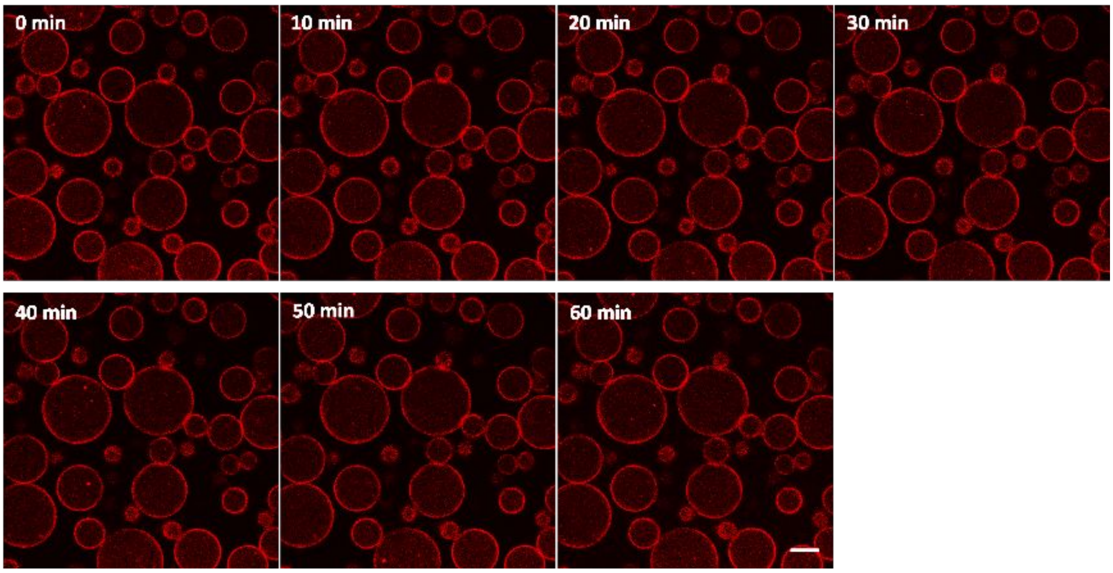

Figure S18. Time series of CLSM images of Au/RITC-TA-AE-PEG nanoparticle-caged coacervate micro-droplets without addition of GOx and glucose. No obvious membrane disassembly was observed within 60 minutes. Scale bar, 20 µm.

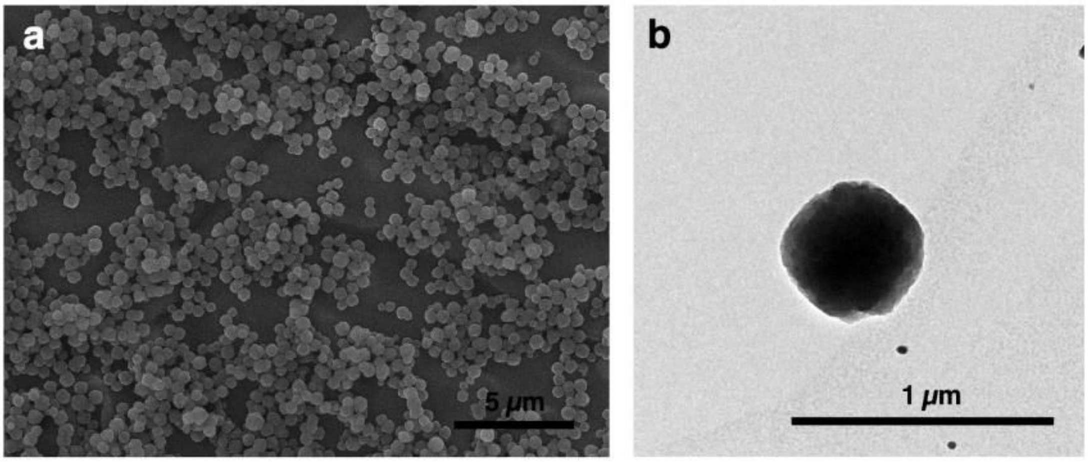

Figure S19. (a) Scanning electron microscopy (SEM) image and transmission electron microscopy (TEM) image showing size and morphology of RITC-labelled BSA@ZIF8 microparticles. Scale bars, 5 µm (a) and 1 µm (b).

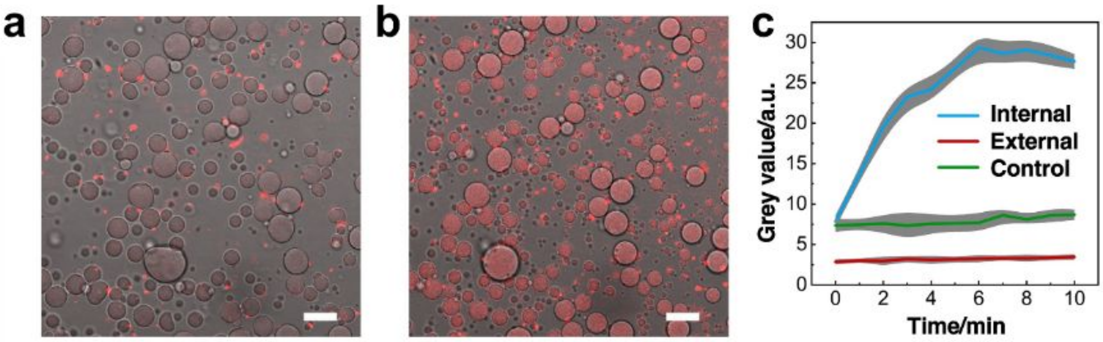

Figure S20. Overlays of bright field and fluorescence CLSM images of an aqueous dispersion of caged Au/TA-PEG coacervate droplets and BSA@ZIF8 microparticles before (**a**) and after 6 minutes of light exposure (**b**) showing triggered uptake into the coacervate interior (red fluorescence); corresponding changes in mean fluorescence recorded inside and outside the caged coacervate droplets, as well as without light illumination (normal daylight, control) are shown in (**c**). Error bars in (**c**) represent the standard deviation (n = 10). Scale bars, 50 µm.

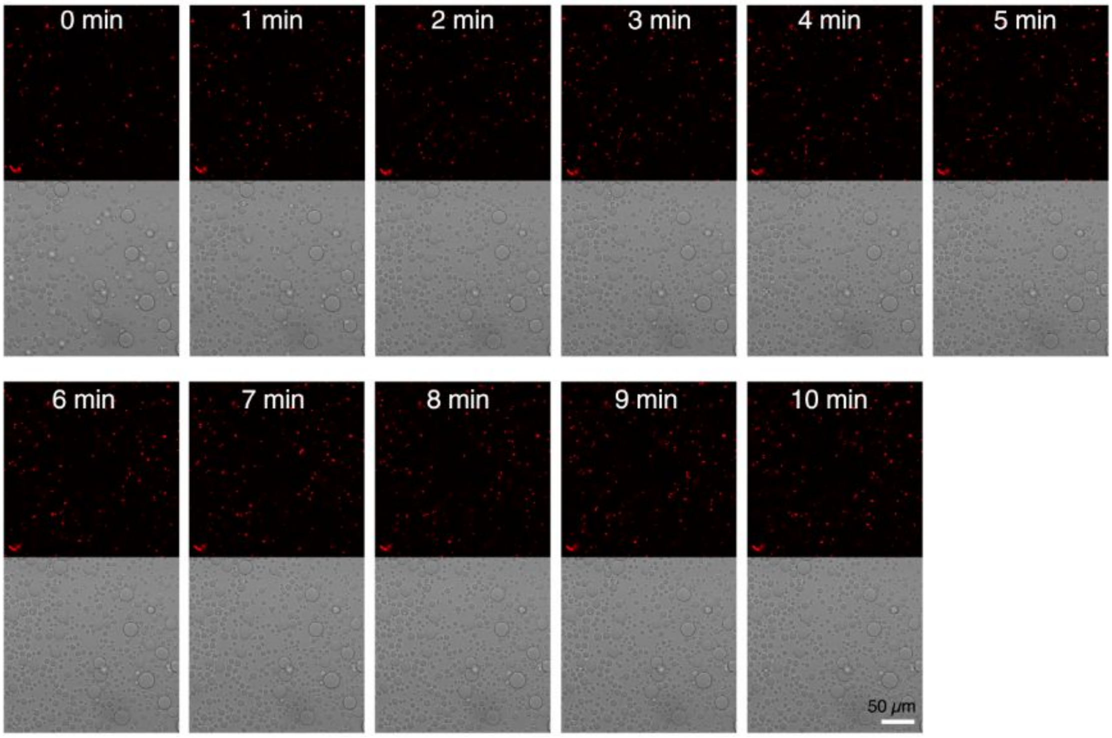

Figure S21. Time series of CLSM images (top: fluorescence field; bottom: bright field) showing no uptake of RITC-labelled BSA@ZIF8 microparticles under ambient daylight in the presence of Au/RITC-TA-PEG nanoparticle-caged coacervate droplets. No changes in the fluorescence images are observed with time. Scale bar, 50 µm.

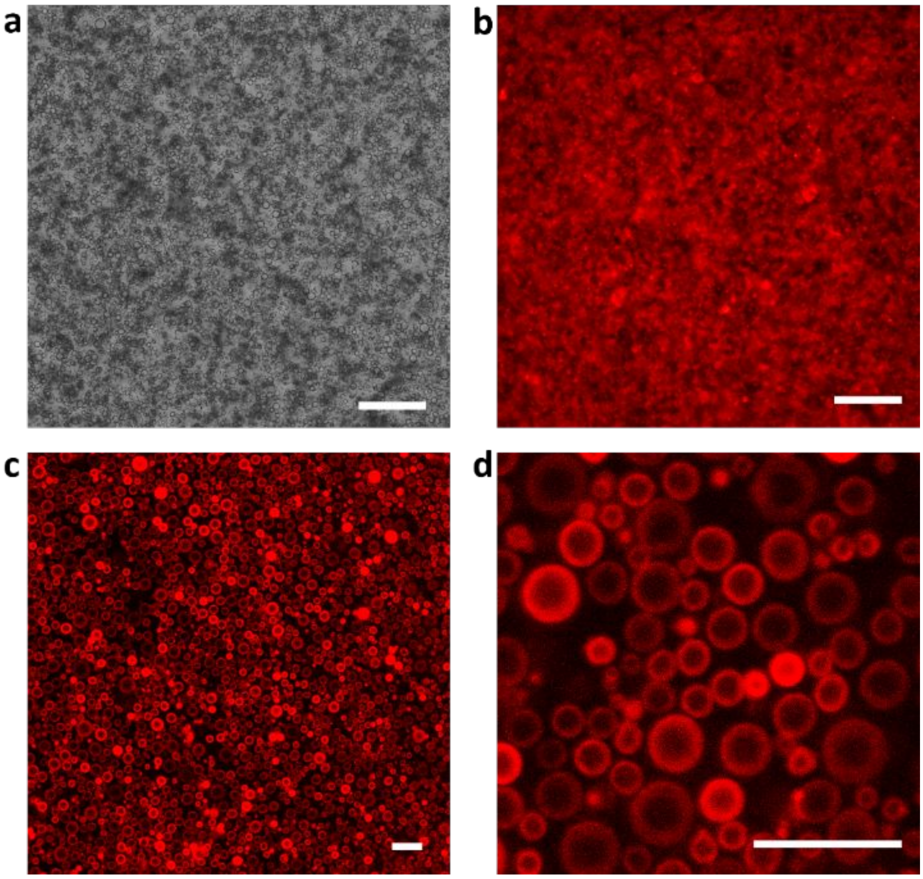

Figure S22. (a) Bright field and (b) fluorescence field microscopy images showing dense populations of PCVs. (c-d) CLSM images showing PCV morphology. Fluorescence is from RITC-labelled amylase encapsulated in the PCVs. Scale bars, 50 µm (a, b) and 10 µm (c, d).

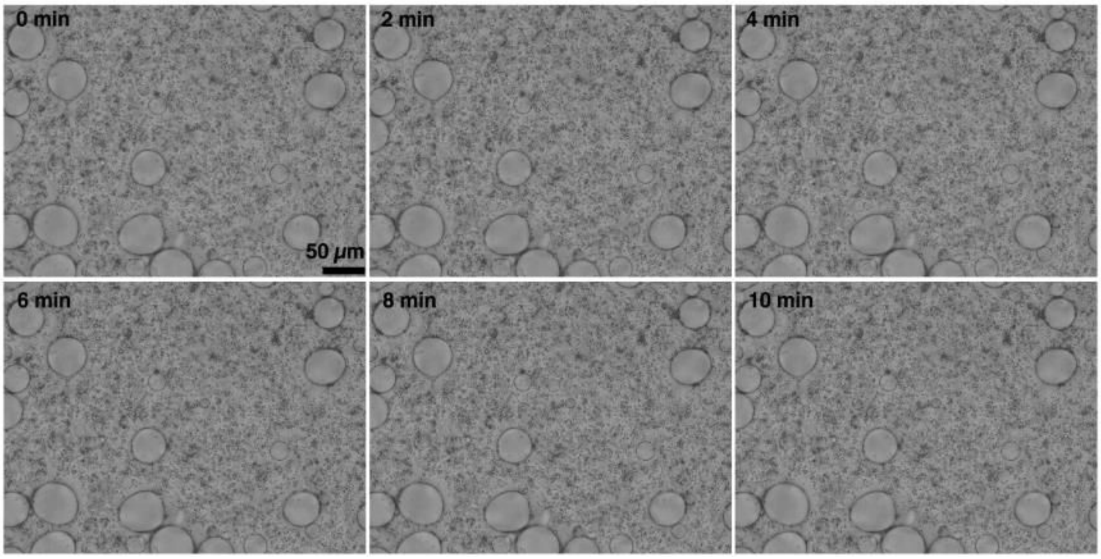

Figure S23. Time series of optical images showing an all-aqueous mixture containing Au/TA-PEG caged coacervate droplets (large objects) and PCVs (small objects) mounted on a pegylated glass slide under ambient daylight (no additional light irradiation). No contact-dependent interactions are observed. Scale bar is 50 µm.

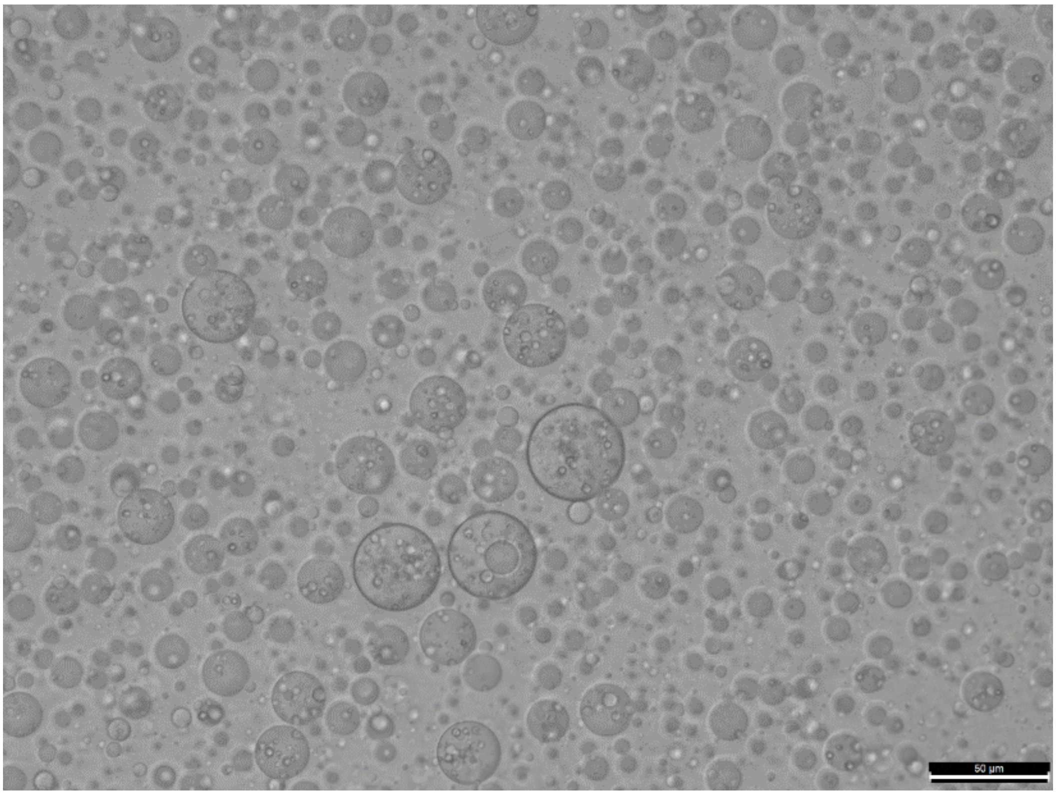

Figure S24. Optical image showing mixture of membrane-less PDDA/CM-dex coacervate microdroplets and PCVs. The PCVs are spontaneously engulfed into the coacervate microdroplets. Scale bar, 50 µm.

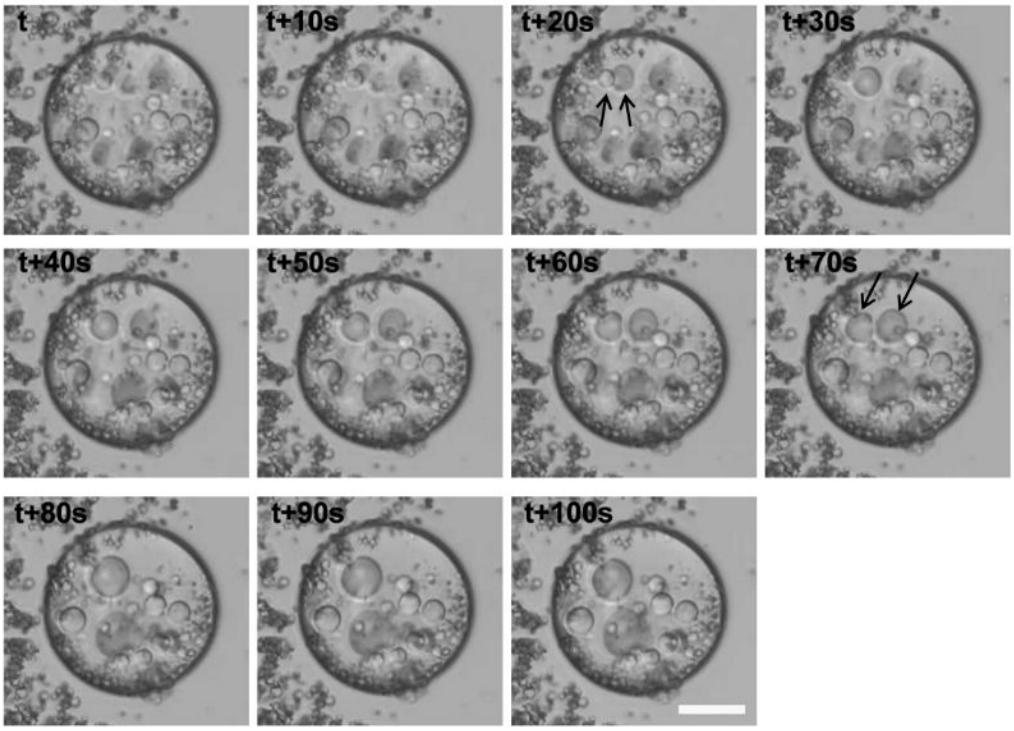

Figure S25. Time series of optical images showing coalescence of PCVs (black arrows) inside a single nanoparticle-caged coacervate droplet. Scale bar, 20 µm.

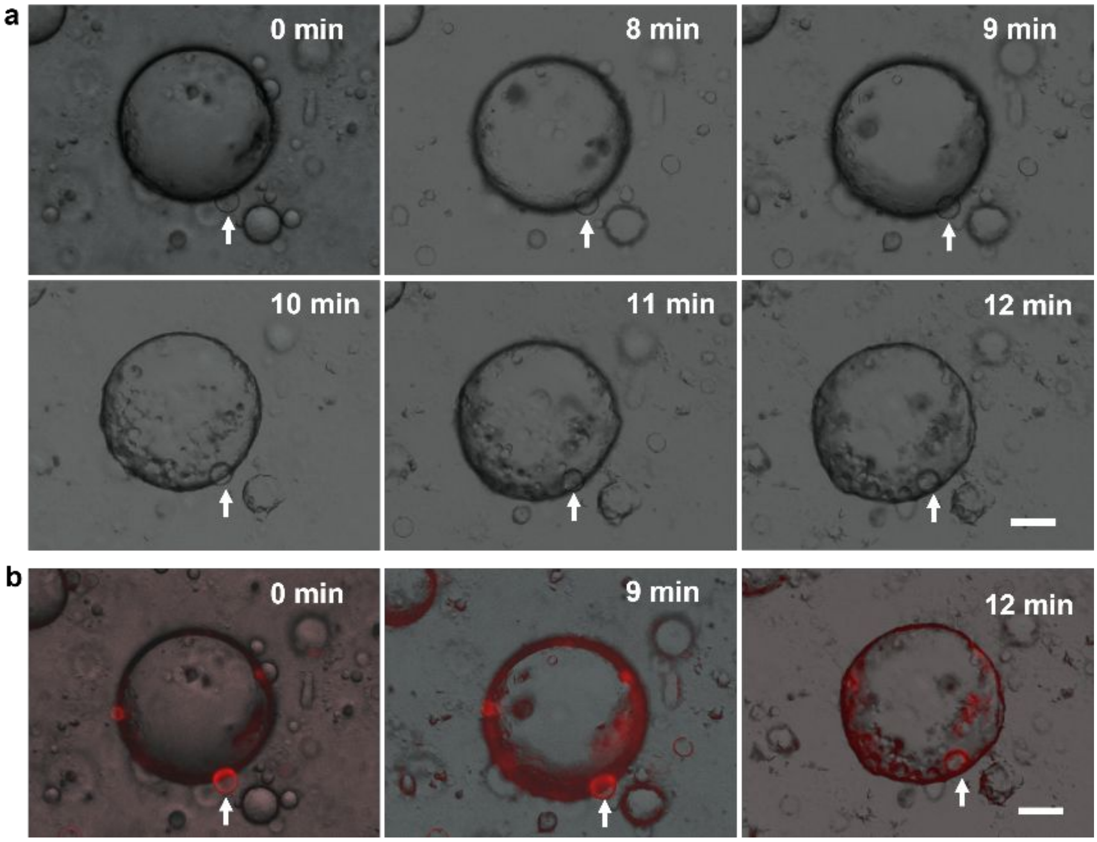

Figure S26. (**a**) Time-series optical images showing capture of a single colloidosome (white arrow) by a caged coacervate droplet. (b) Overlap of the bright field and fluorescence images showing capture of the single RITC-labelled colloidosome. Scale bars, 10 µm.

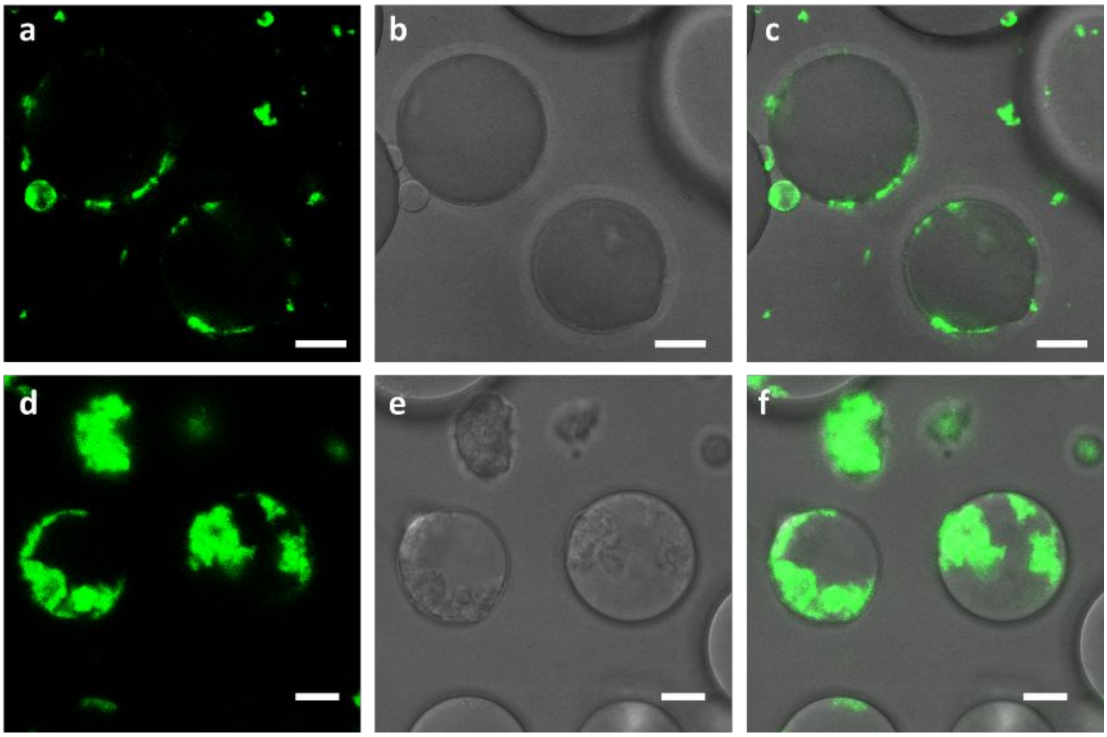

Figure S27. Fluorescence (a,d), bright-field (b,e) and overlay of fluorescence and bright field (c,f) images showing an aqueous suspension of caged coacervate microdroplets and fluorescein-labelled polystyrene nanoparticles before (a-c) and 6 min after (d-f) light illumination showing light-induced capture of the polymer particles. Scale bars, 10 µm.

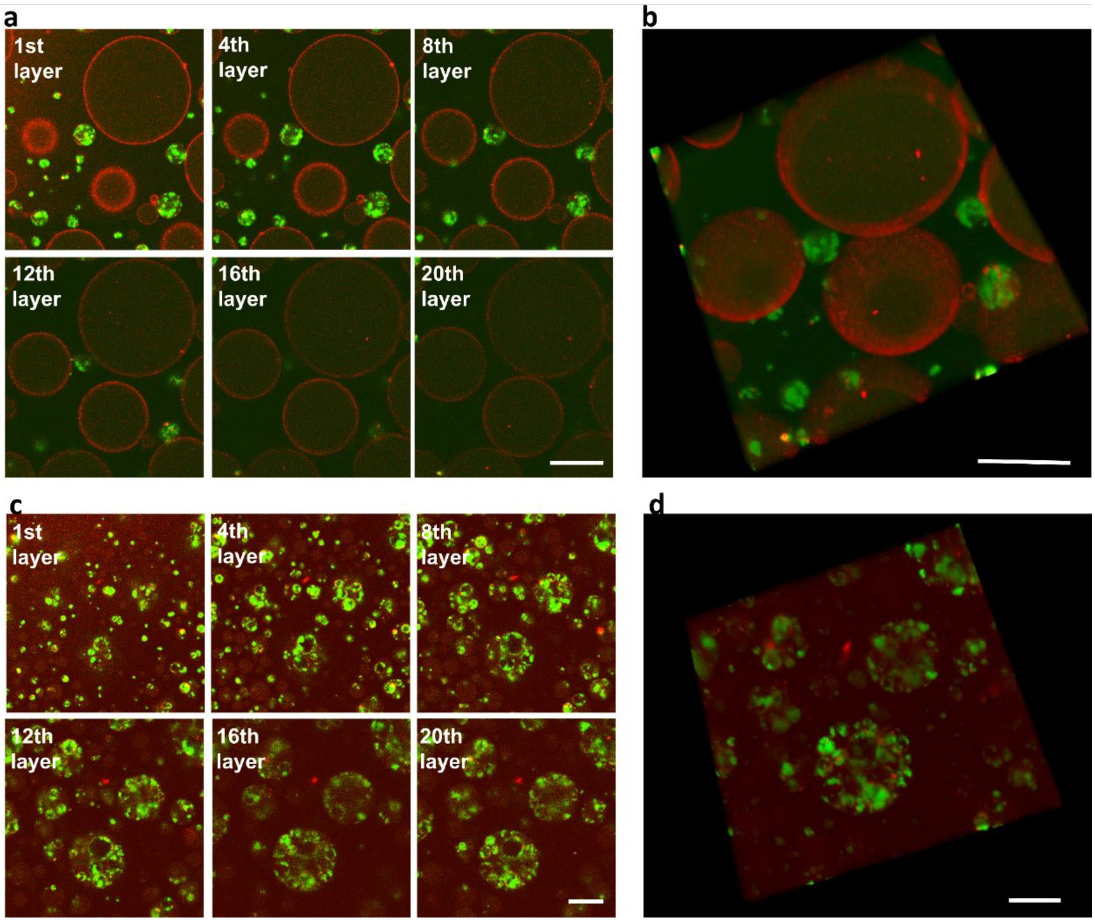

Figure S28. (a, c) CLSM images of red/green fluorescence overlay images showing GOx-containing Au/TA-AE-PEG6k (RITC-labelled membrane, red) caged coacervate droplet surrounded by (FITC-SiO_2)-encapsulated PCVs (green) and recorded before (a) and 60-minutes after (c) addition of glucose. Selected z-direction layers were shown. (b,d) Three-dimensional reconstruction of the CLSM images as shown in (a) and (b), respectively. Scale bars, 20 µm.
